# Commensal *Nakaseomyces glabratus* migrates into prostate tumors to accelerate cancer progression

**DOI:** 10.1038/s43018-026-01229-9

**Published:** 2026-09-09

**Authors:** Ping Lai, Lei Liu, Nicolò Pernigoni, Yingxi Du, Daniele Braga, Martina Troiani, Giuseppe Attanasio, Yuxin Li, Pan Song, Emiliano Pasquini, Tanja Rezzonico Jost, Xiaowen Huang, Martino Maddalena, Simone Mosole, Andrea Rinaldi, Giovanna Pecoraro, Martino Pedrani, Aurora Valdata, Nicolò Bancaro, Luisa Maraccani, Alessandra De Giani, Wei Yuan, Lewis Gallagher, Khobe Chandran, Penny Flohr, Pasquale Rescigno, Giuseppe Reitano, Alessandro Morlacco, Fabrizio Dal Moro, Isabella Giacomini, Monica Montopoli, Matteo Brunelli, Serena Pedron, Ricardo Pereira Mestre, Raad Z. Gharaibeh, Christian Jobin, Silke Gillessen Sommer, Johann de Bono, Andrea Alimonti

**Affiliations:** 1 Institute of Oncology Research (IOR), Bellinzona, Switzerland; 2 Faculty of Biomedical Sciences, Università della Svizzera italiana, Lugano, Switzerland; 3 Medical Center of Hematology, State Key Laboratory of Trauma, Burn and Combined Injury, Xinqiao Hospital, Army Medical University, Chongqing, China; 4 Department of Urology, Institute of Urology, West China Hospital of Sichuan University, Chengdu, China; 5 Oncology Institute of Southern Switzerland (IOSI), Ente Ospedaliero Cantonale (EOC), Bellinzona, Switzerland; 6 Department of Health Sciences and Technology, Eidgenössische Technische Hochschule (ETH) Zürich, Zurich, Switzerland; 7 The Institute of Cancer Research, London, UK; 8 The Royal Marsden Hospital, London, UK; 9 Candiolo Cancer Institute, Turin, Italy; 10 Department of Surgery, Oncology and Gastroenterology, Urologic Unit, University of Padua, Padua, Italy; 11 Department of Pharmaceutical and Pharmacological Sciences, University of Padua, Padua, Italy; 12 Department of Diagnostics and Public Health, University of Verona, Verona, Italy; 13 FISH Molecular Lab, Pathology Unit, Azienda Ospedaliera Universitaria Integrata di Verona (AOUI), Verona, Italy; 14 Department of Medicine, Division of Gastroenterology, Hepatology and Nutrition, University of Florida College of Medicine, Gainesville, FL USA; 15 Department of Molecular Genetics and Microbiology, University of Florida, Gainesville, FL USA; 16 Department of Medicine, University of Padua, Padua, Italy

**Keywords:** Urological cancer, Tumour immunology, Fungi

## Abstract

Recent studies demonstrate that the gut mycobiota plays a key role in several tumors. However, the contribution of commensal fungi to prostate cancer initiation and progression remains understudied. Here we find that *Nakaseomyces glabratus* is enriched in fecal, blood and tumor samples of patients with castration-resistant prostate cancer, correlating with patients’ poor overall survival. Oral administration of *N. glabratus* to castrated mice accelerated cancer progression by promoting infiltration and activation of polymorphonuclear myeloid-derived suppressor cells (PMN-MDSCs). Mechanistically, androgen deprivation therapy increases intestinal permeability, resulting in the leakage of *N. glabratus* from the gut to the tumor site, activating PMN-MDSCs via the Dectin-2 receptor. Treatment of mice with a negatively charged intestinal hydrogel blocks *N. glabratus* translocation to tumor, reducing PMN-MDSCs’ intratumoral infiltration and activation. Taken together, these findings reveal that the gut-to-tumor translocation of *N. glabratus* contributes to endocrine resistance in prostate cancer by enhancing the immunosuppressive microenvironment of these tumors.

## Abstract (web summary)

Lai et al. show that *Nakaseomyces glabratus* is enriched in fecal and tumor samples of patients with castration-resistant prostate cancer and that administration of the fungus accelerates cancer progression in prostate cancer-bearing castrated mice.

## Main

Recent metagenomics analyses have identified the presence of fungi in various solid tumors, including pancreatic, colon, breast and lung cancer^1–3^. Emerging evidence further indicates that the tumor-associated mycobiome plays an active role in cancer progression and therapeutic resistance, sustaining chronic inflammation^4–8^. Patients with prostate cancer are often treated with corticosteroids in combination with anti-androgen therapy and androgen receptor pathway inhibitors (ARPIs), a therapeutic regimen that can increase susceptibility to fungal infections^9^. However, the impact of ADT on the fungal mycobiome and the underlying mechanisms of gut and tumor mycobiota transmission in the progression of prostate cancer (PCa) remain elusive.

Here we detected the presence of *Nakaseomyces glabratus* DNA in the gut, blood and tumor samples of patients affected by PCa. *N. glabratus*—formerly classified as *Candida glabrata*—is an opportunistic yeast that belongs to the *Nakaseomyces* clade, which shares several characteristics with the *Candida* genus^10^. Together with *Candida albicans*, *N. glabratus* is a major cause of nosocomial infection in patients affected by cancer, being also resistant to several clinically used antimycotics^11–13^.

Notably, *N. glabratus* was further found to correlate with poorer survival outcomes in patients with castration-resistant prostate cancer (CRPC). In line with this evidence, oral administration of *N. glabratus* to castrated (CTX) mice promoted cancer progression through the activation of polymorphonuclear myeloid-derived suppressor cells (PMN-MDSCs). It is interesting that we found that androgens contribute to the maintenance of intestinal permeability. Consequently, androgen deprivation therapy (ADT) leads to enhanced gut vascular barrier permeability, facilitating the translocation of intestinal *N. glabratus* into the bloodstream and its subsequent delocalization in prostate tumors. Mechanistically, within the tumor, *N. glabratus* enhances the immunosuppressive and tumor-promoting functions of PMN-MDSCs by activating the Dectin-2–SYK–activin A signaling pathway. Therapeutically, we demonstrated that a negatively charged intestinal hydrogel increases the mouse gut vascular barrier and attenuates tumor growth in castrated prostate tumor-bearing mice by blocking the tumor localization of *N. glabratus* and the subsequent activation of PMN-MDSCs.

## Results

### Commensal *N. glabratus* is detected in fecal samples of patients with PCa

**Fig. 1:**
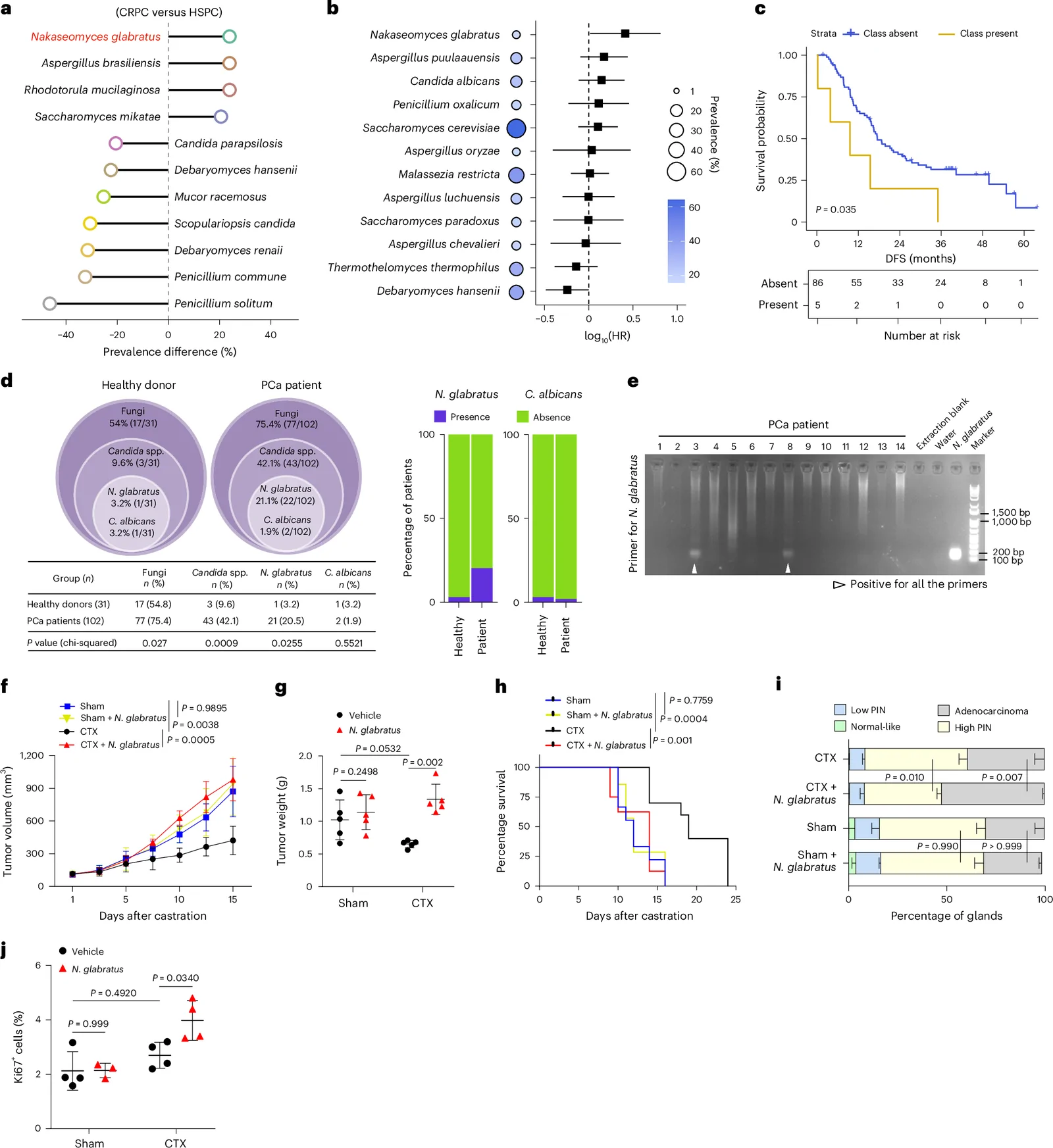
Commensal *N. glabratus* is detected in fecal samples of patients with PCa and promotes prostate tumor growth in vivo. **a**, Waterfall plot representing the prevalence difference of fungi species in patients with CRPC (*n* = 21) and HSPC (*n* = 10) analyzed by ITS sequencing. **b**, Rectal swabs were collected from patients with CRPC (*n* = 91) and shotgun whole-metagenome sequencing was performed. The bubble plot represents the relative prevalence of different fungal species in CRPC. HRs for overall survival by fungal species were estimated using univariate Cox’s regression and are presented as a Forest plot. The *P* values were obtained using Wald’s test. No adjustments were made for multiple comparisons. **c**, Survival analysis on patients with CRPC stratified for the presence or absence of *N. glabratus*. The *P* value is calculated using a log(rank) test. No adjustments were made for multiple comparisons. **d**, Quantitative PCR analysis of fungi, *Candida* spp., *C. albicans* and *N. glabratus* in healthy donors (*n* = 31) and patients with PCa (*n* = 102) showing the absolute number and percentage of positive cases. Positivity of *N. glabratus* was assigned when the amplification signal was detected with the three primer sets (fungi ITS, *Candida* spp. and *N. glabratus*). **e**, Electrophoretic analysis of PCR products amplified with *N. glabratus*-specific primers in fresh-frozen patient tumor samples (*n* = 14; each lane represents 1 individual sample). PCR reactions using *N. glabratus* genomic DNA and water served as positive and negative controls, respectively. **f**, Tumor volume of TRAMP-C1 mice treated with ABX for 7 days before surgery (sham or castration), then orally treated with ABX + *N. glabratus* or ABX. Tumor weight (sham ABX, *n* = 6; sham ABX + *N. glabratus*, *n* = 6; CTX ABX, *n* = 7; and CTX ABX + *N. glabratus*, *n* = 6). All values are presented as the mean ± s.d. The *P* values were calculated on two-way analysis of variance (ANOVA) with Tukey’s multiple-comparison test. **g**, Tumor weight of TRAMP-C1 mice orally treated with ABX + *N. glabratus* or ABX. Each dot represents 1 mouse (sham ABX, *n* = 5; sham ABX + *N. glabratus*, *n* = 5; CTX ABX, *n* = 5; and CTX ABX + *N. glabratus*, *n* = 5). All values are presented as the mean ± s.d. The *P* values were calculated using two-tailed, unpaired Student’s *t*-test with Welch’s correction. **h**, Survival curve of TRAMP-C1 mice treated with ABX for 7 days before surgery (sham or castration), then treated via oral gavage with ABX + *N. glabratus* or ABX (sham ABX, *n* = 9; sham ABX + *N. glabratus*, *n* = 7; CTX ABX, *n* = 10; and CTX ABX + *N. glabratus*, *n* = 8). Kaplan–Meier survival curve statistics were determined using the log(rank) (Mantel–Cox) test. **i**,**j**, Histopathological score of anteroposterior lobes (**i**) and percentage of Ki67-positive cells (**j**) from *Pten*^*pc*^^*−/−*^ mice with *N. glabratus* treatment (sham, *n* = 4; sham + *N. glabratus*, *n* = 3; CTX, *n* = 4; CTX + *N. glabratus*, *n* = 4). The *P* values were calculated using two-way ANOVA with Tukey’s multiple-comparison test (**i**) or with Šidák’s multiple-comparison test (**j**). All values are presented as the mean ± s.d. DFS, disease-free survival; PIN, prostatic intraepithelial neoplasia. Source data

**Extended Data Fig. 1:**
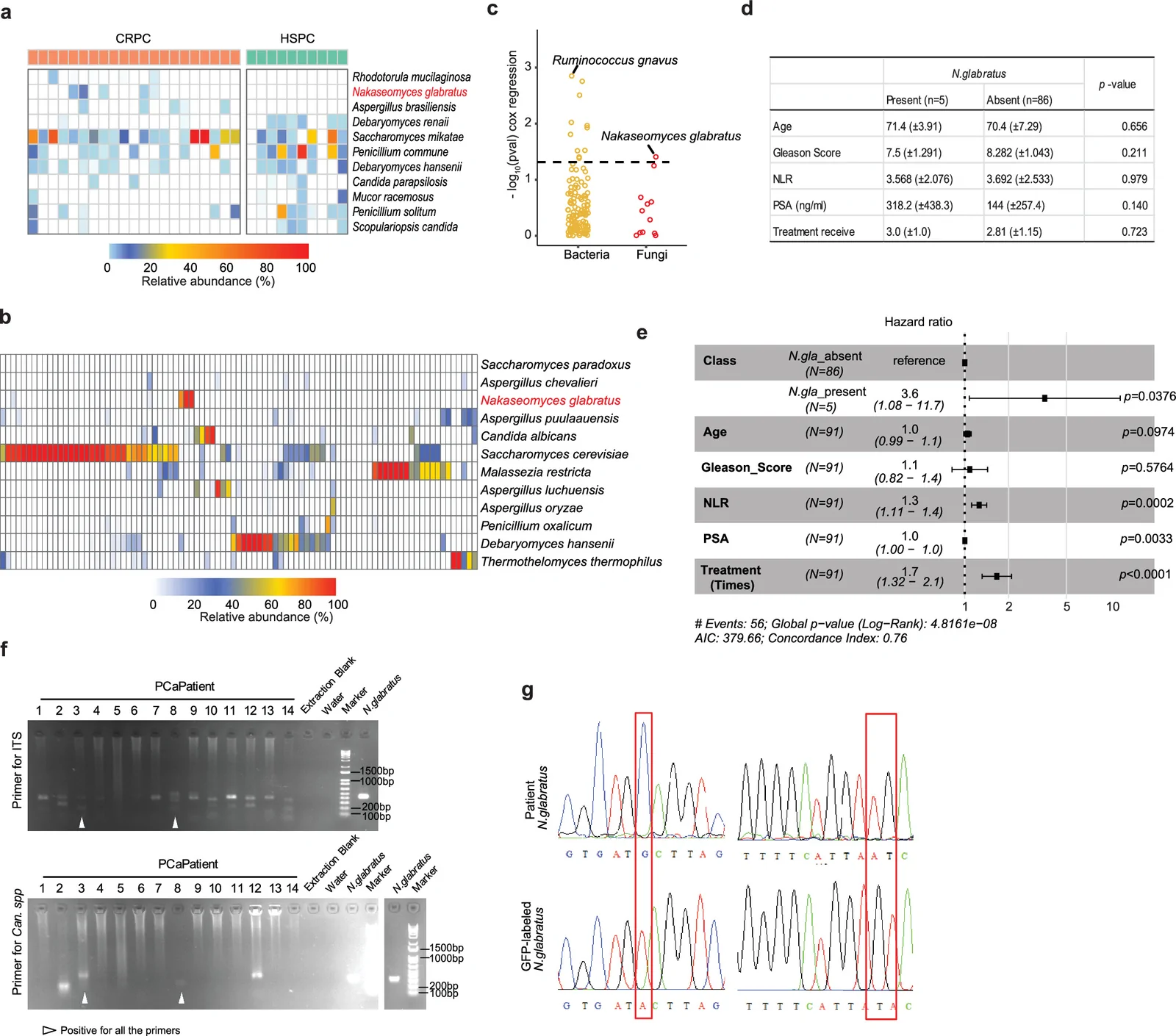
Commensal *N.glabratus* is enriched in CRCP patients. **a:** Heatmap representing differentially abundant fungal species in CRPC (n = 21) and HSPC (n = 10) patients’ fecal samples analyzed by ITS sequencing. **b:** Heatmap representing the relative abundance of fungal species in 91 CRPC patients’ fecal samples analyzed by metagenomics sequencing. **c:** Survival analysis (Univariate cox regression analysis) of bacteria and fungi species, showing significant correlation with OS for *R.gnavus* and *N.glabratus*. The horizontal dashed line shows the significance threshold. *p*-values are obtained with Wald test. No adjustments were made for multiple comparisons. **d:** Demographic and clinical characteristics of *N.glabratus* of patients with fecal samples positive or negative for *N.glabratus*. The *p*-value is calculated by unpaired Mann-Whitney test. **e:** Multivariate Cox-regression analysis showing that *N.glabratus* is significantly associated with worse survival after adjusting for relevant clinical variables. *p*-values for individual coefficients are obtained with Wald test. No adjustments were made for multiple comparisons. **f:** Electrophoretic analysis of PCR products amplified with fungi ITS primer and *Candida spp* primer in fresh-frozen patient tumor samples (n = 14, each lane represents one individual sample.). Of note, patients 3 and 8, which appeared positive for *N.glabratus* primer in Fig. 1e, are also positive for ITS and Candida primers amplification. PCR reactions using *N.glabratus* genomic DNA and water served as positive and negative controls, respectively. **g:** Chromatogram traces showing sequence variations in PCR products (Patient 3 and Patient 8) amplified with *N.glabratus*-specific primers as indicated in Fig. 1e.

Fungi act as necessary commensal or opportunistic pathogens that shape host immunity and expand in immunocompromised or long-term antibiotic-treated or steroid-treated patients, such as those with PCa^14,15^. To assess the gut mycobiota composition in patients with PCa, we collected fecal samples from individuals with hormone-sensitive PCa (HSPC, *n* = 10) and CRPC (*n* = 21). Fungal community profiling was performed using internal transcribed spacer (ITS) sequencing, which offers higher specificity for fungal species identification. Among the different species identified, *N. glabratus*, *Aspergillus brasiliensis* and *Rhodotorula mucilaginosa* were found to be the most enriched fungi in the gut of patients with CRPC (each detected in 23.8% of fecal samples), being undetectable in the gut of patients with HSPC (Fig. 1a and Extended Data Fig. 1a). Metagenomics shotgun sequencing of 91 fecal samples of patients with CRPC confirmed the presence of *N. glabratus* in a small percentage of samples analyzed (Extended Data Fig. 1b). Moreover, *N. glabratus* emerged as the only fungal species significantly associated with a worse patient survival as shown by Cox’s regression analysis (hazard ratio (HR) = 2.6, CI: 1.0–6.5, *P* = 0.042) and Kaplan–Meier curve (*P* = 0.035) (Fig. 1b,c and Extended Data Fig. 1c,d). Multivariate analysis adjusting for age, prior therapies, neutrophil-to-leukocyte ratio, prostate-specific antigen levels and Gleason’s score confirmed that the presence of *N. glabratus* in feces correlates with worse patient survival (Extended Data Fig. 1e). PCR analysis of blood samples of patients with CRPC, using three primer sets, also showed a higher *N. glabratus* DNA prevalence compared with controls (Fig. 1d). On the contrary, *Candida albicans* DNA, a common fungus associated with nosocomial infection, was detected in a small percentage of cases with no difference between healthy donors and patients with PCa (Fig. 1d). Finally, we assessed the presence of *N. glabratus* in 14 fresh-frozen CRPC tumor biopsies. PCR analysis of tumor samples using different primers detected *N. glabratus* DNA in 2 of 14 samples (14.3%) (Fig. 1e and Extended Data Fig. 1f). To exclude potential contamination from the *N. glabratus* strain used in our laboratory, the PCR amplicons obtained from patient samples were subjected to Sanger sequencing. Sequence alignment revealed several nucleotide variations relative to our laboratory strain, thereby ruling out crosscontamination (Extended Data Fig. 1g). Together, these findings provide both experimental and computational evidence supporting the presence of *N. glabratus* in a subset of PCas.

**Extended Data Fig. 2:**
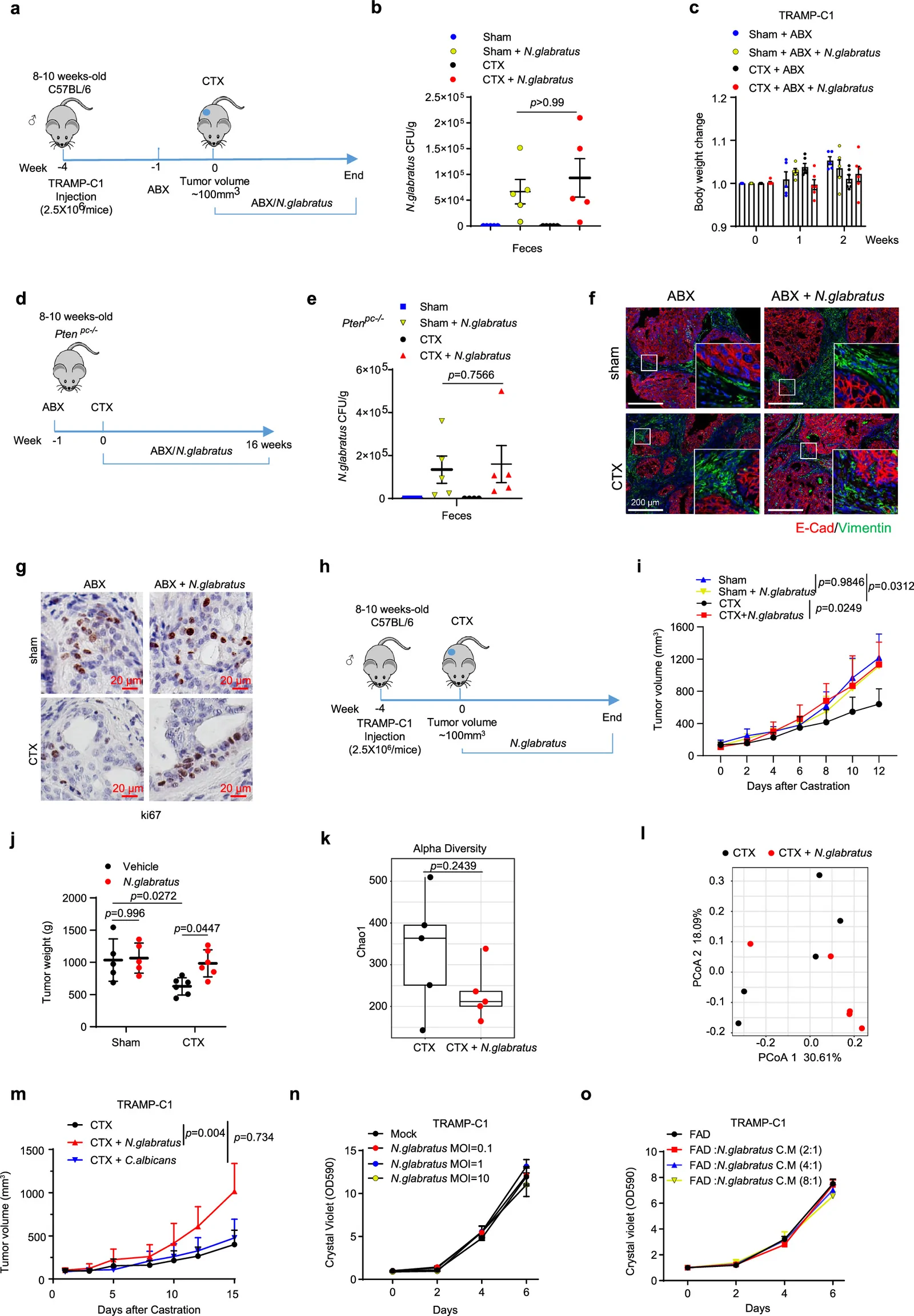
Efficacy of *N.glabratus* treatment in two mouse models of prostate cancer. **a:** Workflow of TRAMP-C1 mice with surgery (sham or castration) and then orally treated with ABX or ABX plus *N.glabratus*. **b:** Colony-forming Unit (CFU) of *N.glabratus* in the feces per grams of TRAMP-C1 mice treated as aforementioned. Each dot represents a value from one mouse (sham, n = 5; sham *N.glabratus*, n = 5; CTX, n = 6; CTX *N.glabratus*, n = 5). *p-*values were calculated using Kruskal-Wallis test with Dunn’s multiple comparisons. **c:** Body weight of TRAMP-C1 mice treated as aforementioned. Each dot represents a value from one mouse (sham, n = 5; sham *N.glabratus*, n = 5; CTX, n = 6; CTX *N.glabratus*, n = 6). *p-*values were calculated using Two-way ANOVA test with Tukey’s multiple comparisons test. **d:** Workflow of *Pten*^*pc*−*/*−^ mice with surgery (sham or castration) and orally treated with ABX or ABX plus *N.glabratus*. *Pten*^*pc–/–*^ mice were sham-operated or castrated at 8-10 weeks old and orally treated with ABX or ABX plus *N.glabratus*. **e:** Colony-forming Unit (CFU) of *N.glabratus* in the feces per grams of CTX *Pten*^*pc*−*/*−^ mice treated as described in (**d**). Each dot represents a value from one mouse (sham, n = 4; sham *N.glabratus*, n = 5; CTX, n = 4; CTX *N.glabratus*, n = 5). *p-*values were calculated using Kruskal-Wallis test with Dunn’s multiple comparisons. **f**, Representative E-Cad/Vimentin IF staining images of *Pten* ^*pc*−*/*−^ mice with surgery (sham or castration) and treated as aforementioned, demonstrating an increased aggressiveness in the treated group of *N.glabratus*; scale bar 200 μm. The image shown is from one representative mouse of three analyzed per group. **g:** Representative Ki67 images of *Pten*^*pc*−*/*−^ mice with surgery (sham or castration) and treated as described in (**d**); scale bar 20 μm. The image shown is from one representative mouse of three analyzed per group. **h:** Workflow of TRAMP-C1 mice with surgery (sham or Castration) and then treated with or without *N.glabratus* under normal microbiome conditions. **i, j:** Tumor volume (**i**) and tumor weight (**j**) of TRAMP-C1 mice treated with or without *N.glabratus*. Each dot represents a value from one mouse (sham, n = 5; sham + *N.glabratus*, n = 5; CTX, n = 6; CTX *N.glabratus*, n = 6). *p-*values were calculated using Two-way ANOVA with Tukey’s multiple comparisons test. **k, l:** Fecal bacterial 16S rDNA was sequenced in *N.glabratus*-treated (CTX + *N.glabratus*) and untreated (CTX) groups (n = 5 mice per group). Chao indexes (**k**) and Principal coordinate analysis (PCoA) (**l**) of bacterial diversity are shown for individual mice. Box plots in (**k**) show the interquartile range (IQR), the horizontal lines show the median values, and the whiskers indicate the minimum-to-maximum range. *p*-value in (**k**) is calculated by one-way ANOVA and controlled for false discovery rate (FDR) by correcting the *p*-values using the Benjamini and Hochberg (BH) approach. **m:** Tumor size of TRAMP-C1 mice treated with ABX, *N.glabratus* or *C.albicans*. CTX, n = 7; CTX plus *N.glabratus*, n = 7; CTX plus *C.abicans*, n = 7. *p-*values were calculated using Two-way ANOVA with Tukey’s multiple comparisons test. **n, o:** Crystal violet staining of TRAMP-C1 cells treated with *N.glabratus* at different MOI (**n**) or *N.glabratus* C.M at different ration (**o**) at indicated time points. Data are representative of one of three independent experiments. All values are presented as the mean ± SD. Source data

We next assessed whether oral administration of *N. glabratus* in prostate tumor-bearing mice could impact PCa progression. To perform this experiment, we used both sham-operated and CTX TRAMP-C1 allografts. Mice were pretreated with a broad-spectrum antibiotic cocktail (ABX) to eradicate the gut bacteria and avoid bacterial or fungal competition for the intestinal niche before performing surgical castration. We found that *N. glabratus* oral administration in mice injected with TRAMP-C1 cancer cells accelerated tumor growth and worsened overall survival in CTX but not in sham-operated mice (Fig. 1f–h and Extended Data Fig. 2a–c). We next examined the impact of *N. glabratus* administration on tumor progression in sham-operated and CTX Pten null prostate conditional (*Pten*^*pc−/−*^) mice, a genetically engineered mouse model that recapitulates human PCa progression from benign prostate tumors to CRPC^16,17^ (Extended Data Fig. 2d,e). Histopathological evaluation of the anterior prostate lobes of *Pten*^*pc*−*/*−^ CTX mice supplemented with *N. glabratus* showed a marked increase in tumor aggressiveness and percentage of Ki67-positive cells compared with sham-operated mice treated with and without *N. glabratus* (Fig. 1i,j and Extended Data Fig. 2f,g). In the absence of ABX treatment, *N. glabratus* administration to CTX TRAMP-C1 allografts still promoted tumor cell proliferation, demonstrating that the tumor-promoting effect of *N. glabratus* is independent of its impact on the bacterial microbiota (Extended Data Fig. 2h–l). It is interesting that *C. albicans* oral administration to CTX TRAMP-C1-bearing mice had no notable influence on tumor growth, validating the species-specific effects of *N. glabratus* on tumor growth and progression (Extended Data Fig. 2m). We next assessed whether *N. glabratus* could directly affect tumor cell proliferation in vitro. Neither *N. glabratus* nor *N. glabratus-*treated conditioned medium (CM) promoted TRAMP-C1 cell growth in vitro (Extended Data Fig. 2n,o). Overall, these data show that *N. glabratus* is enriched in the gut of patients affected by PCa and that *N. glabratus* administration to tumor-bearing mice drives cancer progression in both ABX-treated and untreated mice.

### *N. glabratus* administration increases PMN-MDSC infiltration and immunosuppressive function

**Fig. 2:**
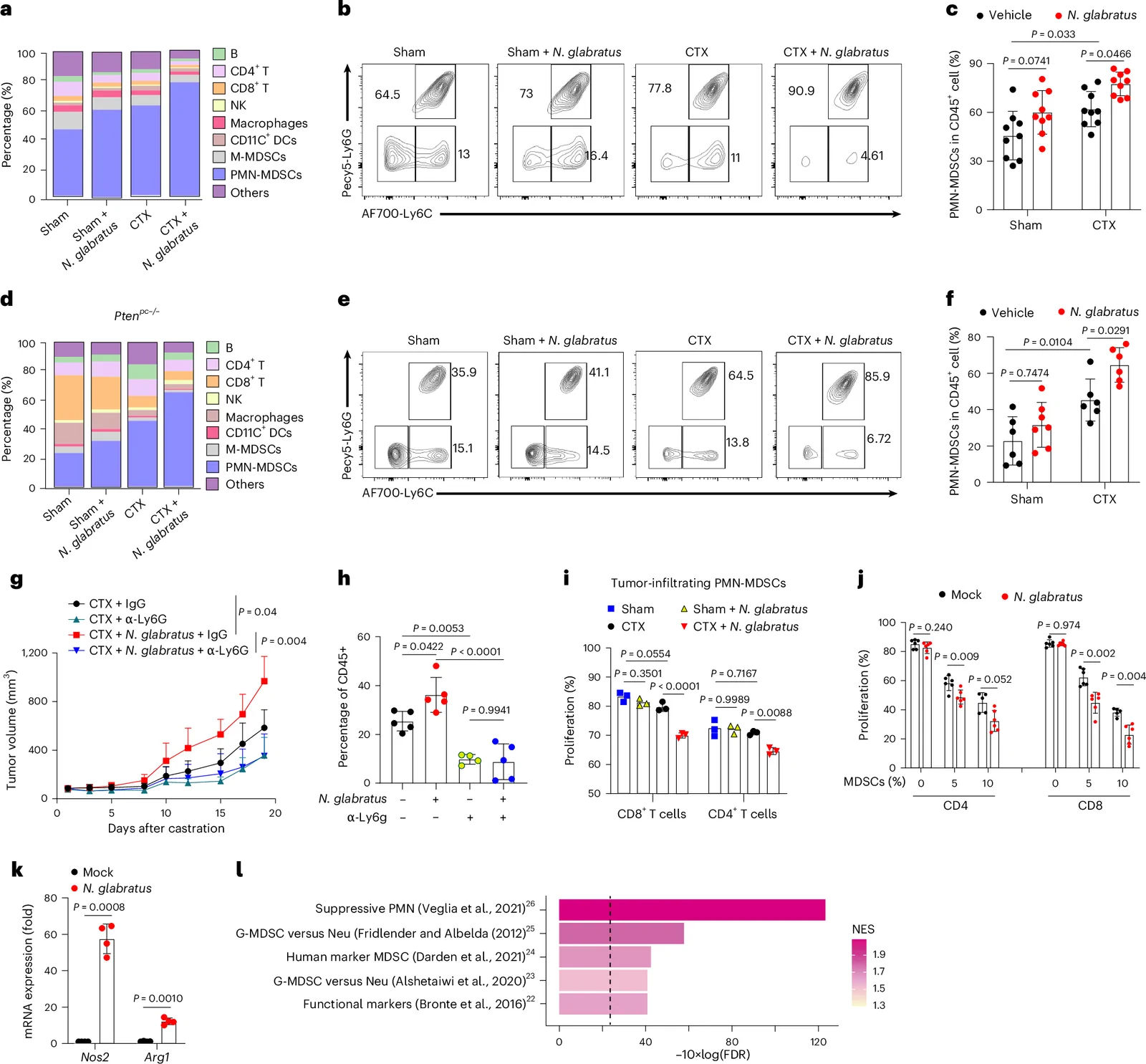
*N. glabratus* administration increases PMN-MDSC infiltration with enhanced immunosuppressive function. **a**, Percentages of tumor-infiltrating immune cell populations (gated on CD45^+^ cells) of TRAMP-C1 tumor-bearing mice CTX with ABX or ABX + *N. glabratus* (sham ABX, *n* = 9; sham ABX + *N. glabratus*, *n* = 9; CTX ABX, *n* = 9; and CTX ABX + *N. glabratus*, *n* = 9). **b**, Representative FACS images of CD45^+^CD11b^+^-gated immune cell subpopulations in TRAMP-C1 tumor-bearing mice with ABX or ABX + *N. glabratus*. The values represent the percentage of cells within the parent population (CD45^+^CD11b^+^). **c**, Percentages of tumor-infiltrating PMN-MDSCs (gated on CD45^+^ cells) of the experiment as mentioned in **a**. The *P* values were calculated using two-way ANOVA with Šidák’s multiple-comparison test. **d**, Percentages of tumor-infiltrating immune populations (gated on CD45^+^ cells) of *Pten* ^*pc*−*/*−^ mice with surgery (sham or castration) and treated with ABX or ABX + *N. glabratus* (sham ABX, *n* = 6; sham ABX + *N. glabratus*, *n* = 6; CTX ABX, *n* = 5; and CTX ABX + *N. glabratus*, *n* = 5). **e**, Representative FACS images of immune cell subpopulations (CD45^+^CD11b^+^ gated) in *Pten*^*pc*−*/*−^ mice with surgery (sham or castration) and then treated with ABX or ABX + *N. glabratus* as mentioned in **d**. **f**, Percentages of tumor-infiltrating PMN-MDSCs (gated on CD45^+^ cells) of the experiment mentioned in **d**. The *P* values were calculated using two-way ANOVA with Šidák’s multiple-comparison test. **g**, Tumor size of TRAMP-C1 tumor-bearing mice treated with CTX + ABX or CTX + ABX + *N. glabratus*, with or without α-Ly6G-mediated depletion of PMN-MDSCs (CTX ABX + IgG, *n* = 5; CTX ABX + α-Ly6G, *n* = 4; CTX ABX + *N. glabratus* and IgG, *n* = 5; and CTX ABX + *N. glabratus* and α-Ly6G, *n* = 5). The *P* values were calculated using two-way ANOVA with Tukey’s post-hoc test. **h**, Percentages of tumor-infiltrating PMN-MDSCs of the experiment mentioned earlier. Data pooled from each dot represent the value from 1 mouse (CTX ABX + IgG, *n* = 5; CTX ABX + α-Ly6G, *n* = 4; CTX ABX + *N. glabratus* and IgG, *n* = 5; and CTX ABX + *N. glabratus* and α-Ly6G, *n* = 5). The *P* values were calculated using one-way ANOVA with Tukey’s post-hoc test. **i**, Suppression assay of T cell proliferation by CD11b^+^Ly6C^int^Ly6G^+^ PMN-MDSCs sorted from sham and CTX TRAMP-C1 tumor-bearing mice with or without *N. glabratus* administration (*n* = 3 biological samples per group). The *P* values were calculated using one-way ANOVA with Tukey’s correction for multiple comparisons. **j**, Quantification of suppression assay of T cell proliferation by BM-MDSCs treated with *N. glabratus* versus mock control. Each dot represents a single experiment (*n* = 6 per group). The *P* values were calculated using two-tailed Mann–Whitney *U*-test with Holm–Šidák multiple comparison. **k**, RT–qPCR analysis of *Nos2* and *Arg1* expression in BM-MDSCs treated with *N. glabratus*. Each dot represents an experiment (*n* = 4 per group). The *P* values were calculated using two-tailed, unpaired Student’s *t*-test with Welch’s correction for Holm–Šidák multiple comparisons. **l**, Bioinformatics analysis of immunosuppressive markers in BM-MSDCs treated with *N. glabratus*. The bar plots show the enrichment of immunosuppressive signature in BM-MDSCs treated with *N. glabratus* by gene set enrichment analysis (*n* = 3 biological samples per group)^22–26^. The bar color describes the normalized enrichment score (NES), whereas the bar length shows the level of statistical significance. All values are presented as the mean ± s.d. DC, dendritic cell; FDR, false recovery rate; G-MDSC, granulocytic MDSC (also known as PMN-MDSC); IgG, immunoglobulin G; M-MDSC, monocytic MDSC; NK, natural killer cell; Neu, neutrophils. Source data

**Extended Data Fig. 3:**
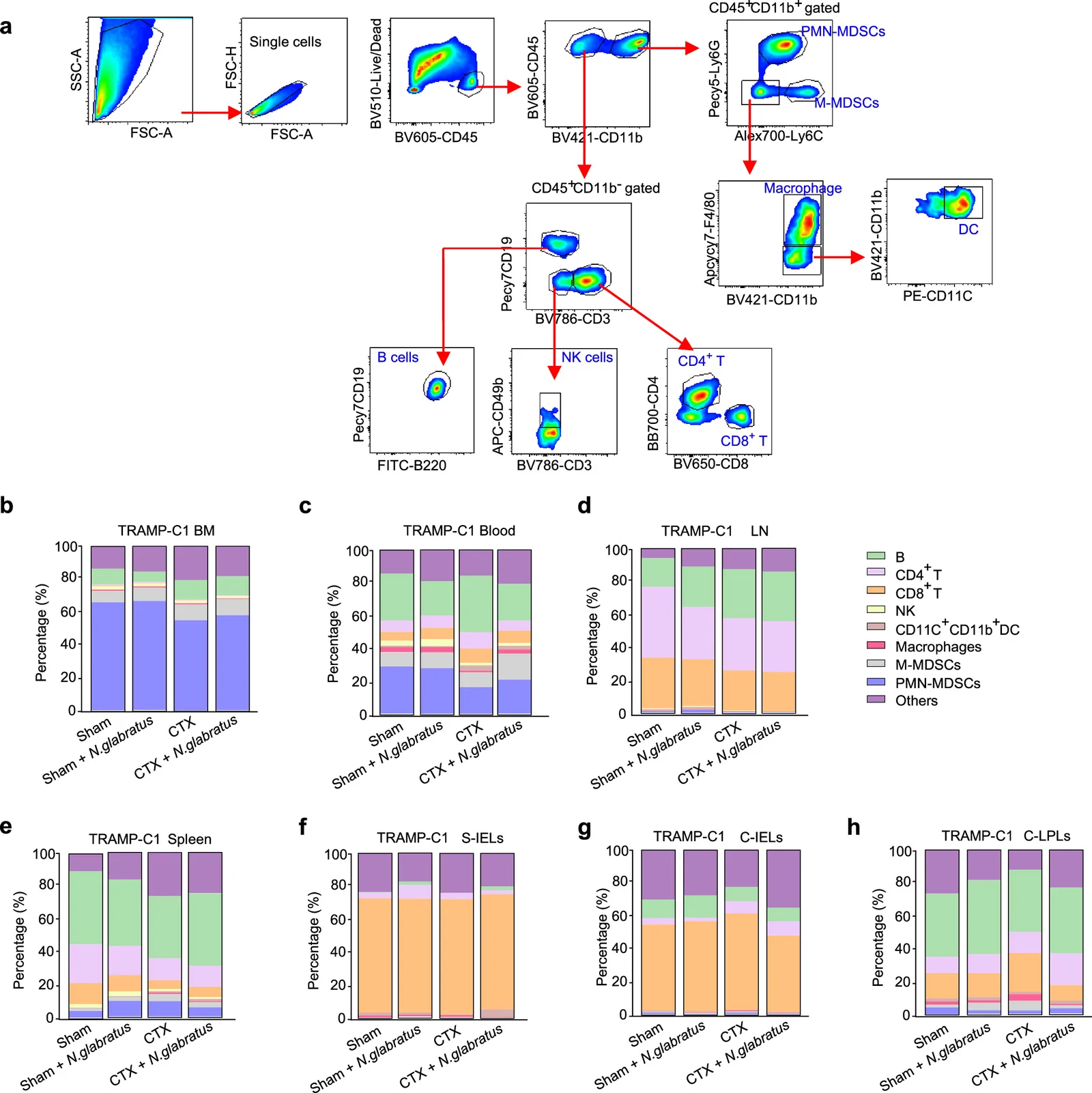
Commensal fungal *N.glabratus* has no impact on immune cell infiltration of other immune organs in TRAMP-C1 model. **a**: Gating strategy of immune cell populations in the tumor microenvironment. The gating strategy is applied in the same way in all the other figures unless otherwise specified. **b-h:** Percentages of immune cell populations (gated on CD45+ cells) in TRAMP-C1 tumor-bearing mice treated with ABX for 7 days before surgery (sham or castration), then treated via oral gavage with ABX plus *N.glabratus* or ABX. Sham ABX (n = 5), CTX ABX (n = 4), sham ABX plus *N.glabratus* (n = 4), and CTX ABX plus *N.glabratus* (n = 5) on Bone marrow (BM) (**b**), Blood (**c**), Lymph nodes (LN) (**d**), Spleen (**e**), Small intestinal intraepithelial lymphocytes (S-IELs) (**f**), Colonic intraepithelial lymphocytes (C-IELs) (**g**), Colonic lamina propria lymphocytes (**h**). Source data

**Extended Data Fig. 4:**
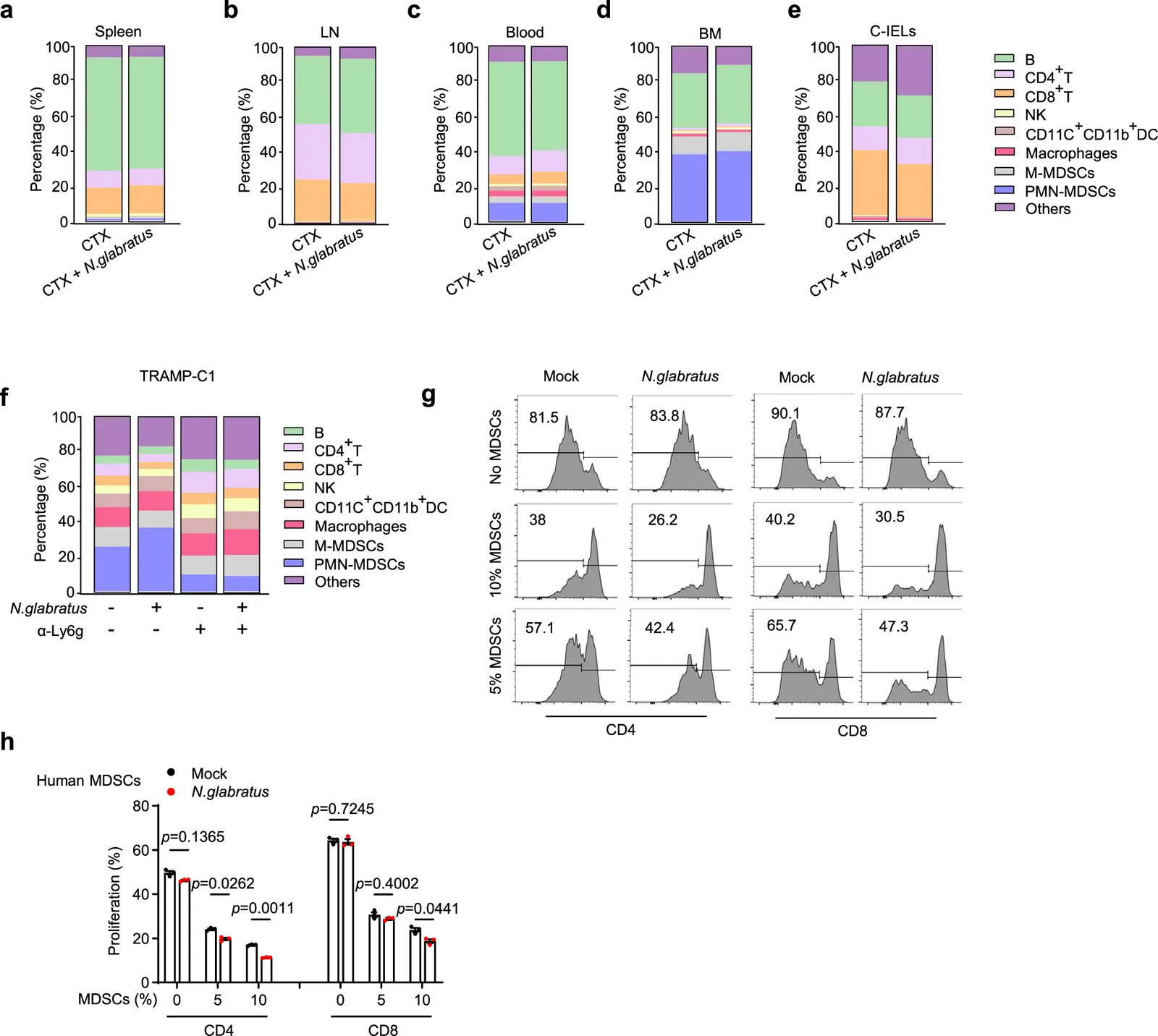
Commensal fungal *N.glabratus* has no impact on immune cell infiltration of other immune organs in CTX *Pten*^*pc*−*/*−^ model. **a-e:** Percentages of tumor-infiltrating immune cell populations (gated on CD45+ cells) of *Pten*^*pc*−*/*−^ mice treated with ABX for 7 days before surgery (castration), then treated via oral gavage with ABX plus *N.glabratus* or ABX. CTX ABX (n = 5) and CTX ABX plus *N.glabratus* (n = 5) on Spleen (**a**), Lymph nodes (LN) (**b**), Blood (**c**), Bone Marrow (BM) (**d**), and Colonic intraepithelial lymphocytes (C-IELs) (**e**) in *Pten*^*pc*−*/*−^ mice. **f:** Percentages of tumor-infiltrating immune cells of TRAMP-C1 tumor-bearing mice treated with CTX + ABX or CTX + ABX + *N.glabratus*, with or without α-Ly6G–mediated depletion of PMN-MDSCs. (CTX ABX plus IgG, n = 5; CTX ABX plus α-Ly6G, n = 4; CTX ABX plus *N.glabratus* and IgG, n = 5; CTX ABX plus *N.glabratus* and α-Ly6G, n = 5). **g:** Representative FACS analyses of suppression assay of T cell proliferation by BM-MDSCs treated with *N.glabratus*. MDSC and T cells were seeded at a ratio of 5% or 10%. The images shown are representative of one of three independent experiments. **h:** Quantification of suppression assay of T cell proliferation by human MDSCs treated with *N.glabratus*. Each dot represents a value from one independent experiment (n = 3 per group). *p*-values were calculated using Two-way ANOVA with Tukey’s multiple comparisons test. All values are presented as the mean ± SD. Source data

Recent evidence indicates that commensal fungi play an active role in shaping host immune responses, thereby contributing to tumor development and modulating the efficacy of anti-cancer therapies^4,5,8,18^. To investigate whether *N. glabratus* promotes PCa progression in relation to immune response, we characterized the immune landscape of prostate tumor-bearing mice supplemented with and without *N. glabratus*. In both TRAMP-C1 and *Pten*^*pc*−*/*−^ tumors treated with *N. glabratus*, we found a significant increase in tumor-infiltrating CD11b^+^Ly6G^+^Ly6C^int^ PMN-MDSCs (also defined as immunosuppressive neutrophils), but not in other immune cell subsets, compared with untreated mice (Fig. 2a–f, Extended Data Fig. 3a and Supplementary Fig. 1). In contrast, *N. glabratus* administration in the same mice did not change the percentage of PMN-MDSCs in normal organs (Extended Data Figs. 3b–h and 4a–e). Previous evidence from our team and others has shown that PMN-MDSCs can promote androgen-independent prostate tumor progression^19–21^. Therefore, we administered *N. glabratus* to CTX mice and subsequently treated these mice with an anti-Ly6G antibody to deplete PMN-MDSCs. *N. glabratus* oral administration increased tumor growth in CTX mice, but PMN-MDSC elimination by anti-Ly6G antibody treatment reversed this effect (Fig. 2g,h and Extended Data Fig. 4f), suggesting that *N. glabratus* promotes tumor growth through PMN-MDSCs. We then evaluated the immunosuppressive capacity of PMN-MDSCs in mice supplemented with *N. glabratus* by analyzing the proliferation of splenic T cells co-cultured with CD11b^+^Ly6G^+^Ly6C^int^ cells sorted from TRAMP-C1 tumors. This analysis revealed that *N. glabratus* administration to tumor-bearing mice enhanced the ability of intratumoral PMN-MDSCs to suppress T cell proliferation ex vivo (Fig. 2i). In line with this result, *N. glabratus* supplementation also enhanced the immunosuppressive capability of both mouse bone marrow-derived MDSCs (BM-MDSCs) (Fig. 2j and Extended Data Fig. 4g) and human-derived MDSCs (hMDSCs) in vitro (Extended Data Fig. 4h). Quantitative PCR with reverse transcription (RT–qPCR) analysis in BM-MDSCs treated with *N. glabratus* showed increased expression levels of immune suppressive genes, such as *Nos2* and *Arg1* (Fig. 2k). This was confirmed by RNA sequencing (RNA-seq) and bioinformatics analysis using different immunosuppressive gene signatures^22–26^ in BM-MDSCs treated with *N. glabratus* in vitro (Fig. 2l). Taken together, these data indicate that administration of *N. glabratus* in CTX prostate tumor-bearing mice induces the recruitment and enhances the immunosuppressive function of PMN-MDSCs in the prostate tumor microenvironment (TME).

### *N. glabratus* activates PMN-MDSCs via Dectin-2

**Fig. 3:**
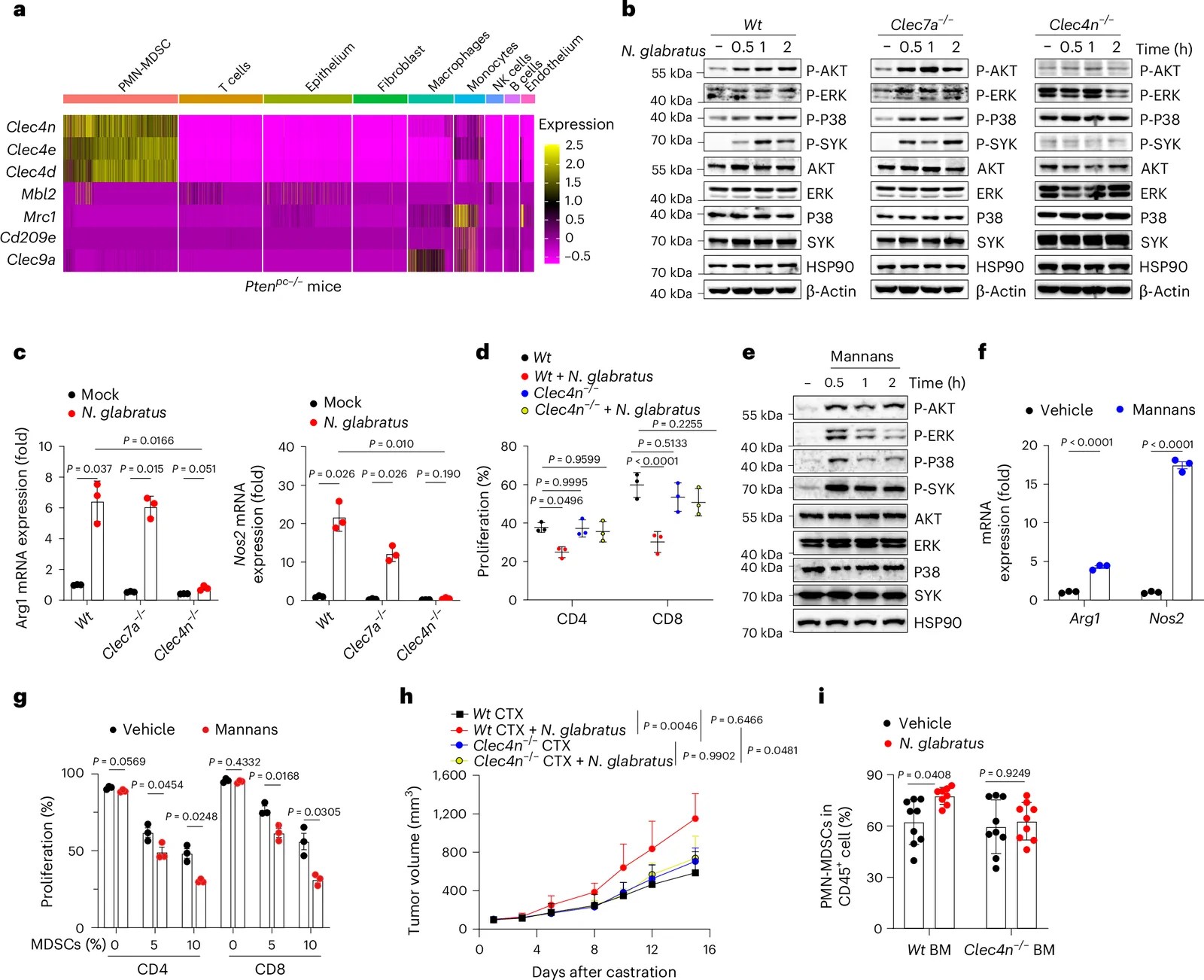
*N. glabratus* activates and primes PMN-MDSCs through Dectin-2-mediated recognition. **a**, Heatmap from scRNA-seq analysis showing the expression level of fungal receptors in different cell types present in the tumor of *Pten*^*pc*−*/*−^ mice. Prostate lobes from two CTX *Pten*^*pc*−*/*−^ mice were pooled equally for scRNA-seq. **b**, Western blotting analysis of WT, Dectin-1^−/−^ and Dectin-2^−/−^ BM-MDSCs treated with *N. glabratus* (multiplicity of infection (MOI) = 5) for the indicated timepoints. The cell lysates were subjected to western blotting analysis using the indicated antibodies. Data represent one of three independent experiments. **c**, RT–qPCR analysis of the indicated gene expression in WT, Dectin-1^−/−^ and Dectin-2^−/−^ BM-MDSCs treated with *N. glabratus* (MOI = 5) for 12 h. Each dot represents an independent experiment (*n* = 3 per group). The *P* values were calculated using two-tailed, unpaired Student’s *t*-test with Welch’s correction for Holm–Šidák multiple comparisons. **d**, Suppression assay of T cell proliferation of *Wt* and *Clec4n*^−*/*−^ BM-MDSCs treated with *N. glabratus*. Each dot represents an independent experiment (*n* = 3 per group). The *P* values were calculated using two-way ANOVA with Tukey’s post-hoc test. **e**, Western blotting analysis of signaling pathway activation in BM-MDSCs stimulated with mannans at different times. Data represent one of three independent experiments. **f**, RT–qPCR analysis of *Nos2* and *Arg1* expression in BM-MDSCs treated with mannans. Each dot represents an independent experiment (*n* = 3 per group). The *P* values were calculated using two-tailed, unpaired Student’s *t*-test with Holm–Šidák multiple comparisons. **g**, Quantification of suppression assay of T cell proliferation by BM-MDSCs treated with mannans. Each dot represents an independent experiment (*n* = 3 per group). The *P* values were calculated using two-tailed, unpaired Student’s *t*-test with Welch’s correction. **h**,**i**, Tumor size (**h**) and tumor-infiltrating PMN-MDSCs (**i**) of TRAMP-C1 tumor-bearing *Wt* and *Clec4n*^−*/*−^ mice that are CTX and orally given ABX or ABX + *N. glabratus* (*Wt* CTX, *n* = 7; *Wt* CTX + *N. glabratus*, *n* = 7; *Clec4n*^−*/*−^ CTX, *n* = 6; and *Clec4n*^−*/*−^ CTX + *N. glabratus*, *n* = 6). Each dot represents one mouse. The *P* values were calculated using two-way ANOVA with Tukey’s multiple-comparison test (**h**) or Šidák’s multiple comparison (**i**). All values are presented as the mean ± s.d. Source data

**Extended Data Fig. 5:**
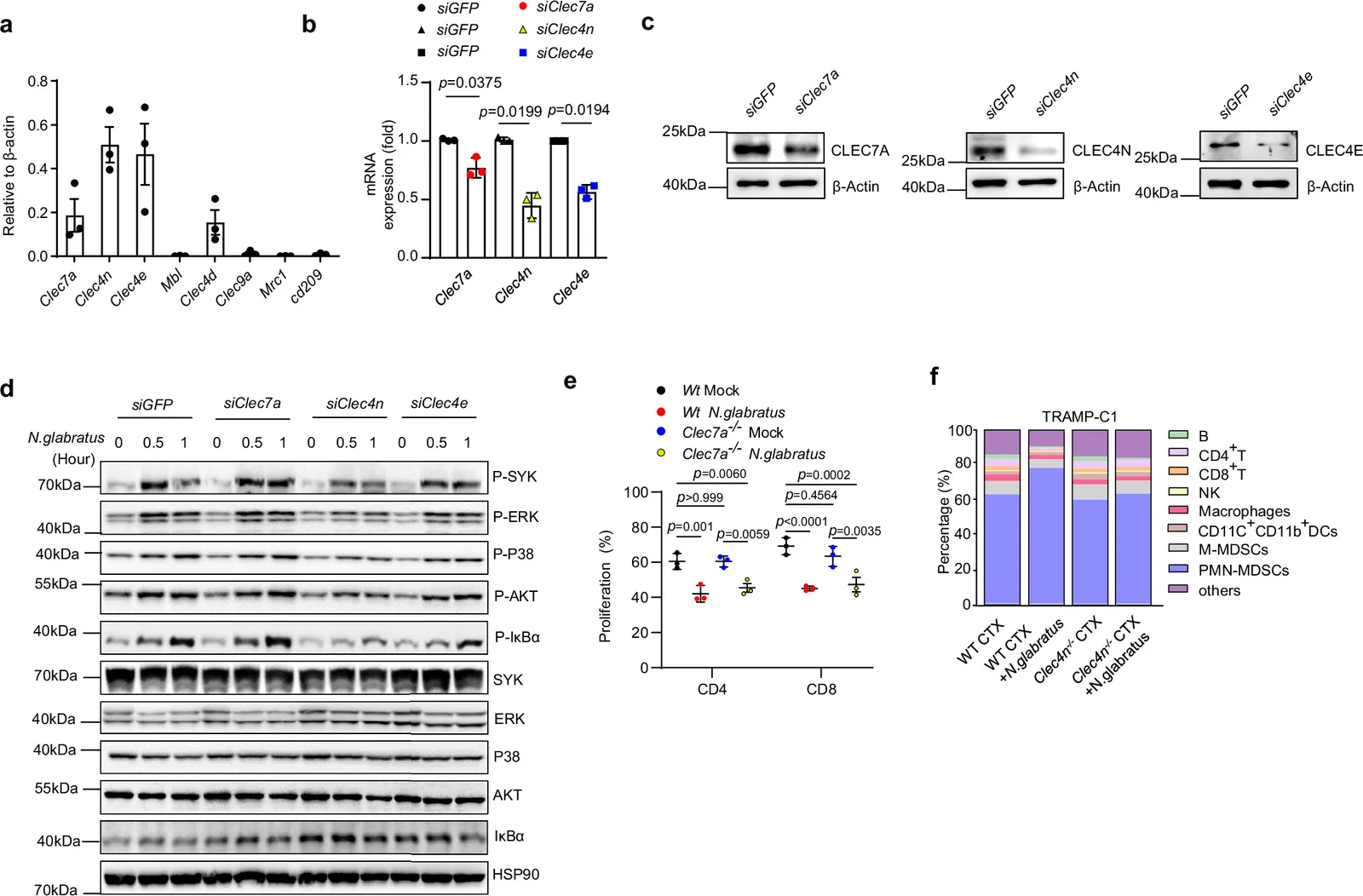
*N.glabratus* drives MDSCs activation via Dectin-2 pathway. **a**: RT-qPCR analysis of the indicated genes in PMN-MDSCs sorting from TRAMP-C1 tumor-bearing mice. Each dot represents a value from one mouse (n = 3 per group). **b:** RT-qPCR analysis of knockdown efficacy of siRNA interference in BM-MDSCs. Each dot represents a value from one independent experiment (n = 3 per group). *p-*values were calculated using two-tailed unpaired Welch t test with Holm-Sidak’s multiple comparisons. **c:** Western blot analysis of CLEC7A, CLEC4N, and CLEC4E expression in BM-MDSCs with siRNA interference using different siRNA as indicated. *siGfp* was used as a negative control. Representative immunoblots from one of three independent experiments are shown. **d:** Western blot analysis of signaling pathway activation in BM-MDSCs with siRNA interference (*siGfp*, *siClec7a, siClec4n* and *siClec4e*) and stimulated with or without *N.glabratus*. Representative immunoblots from one of three independent experiments are shown. **e:** Immunosuppressive activity of *Wt* and *Clec7a*^−*/*−^ BM-MDSCs treated with *N.glabratus*. Each dot represents a value from one independent experiment (n = 3 per group). *p*-values were calculated using Two-way ANOVA with Tukey’s multiple comparisons test. **f:** Tumor-infiltrating immune populations of TRAMP-C1 tumor-bearing *Wt* and *Clec4n*^−*/*−^ mice with CTX and orally given ABX or ABX plus *N.glabratus*. (*Wt* CTX, n = 7; *Wt* CTX + *N.glabratus*, n = 7; *Clec4n*^−*/*−^ CTX, n = 6; *Clec4n*^−*/*−^ CTX + *N.glabratus*, n = 6). All values are presented as the mean ± SD. Source data

Next, we sought to elucidate the mechanism by which *N. glabratus* enhances MDSC function in vivo. Carbohydrate-rich molecules on the fungal cell surface can engage C-type lectin receptors (CLRs) that can be expressed by various immune cells, thereby triggering antifungal immune responses. Among the key CLRs are Dectin-1 (encoded by *Clec7a*), Dectin-2 (encoded by *Clec4n*) and Mincle (encoded by *Clec4e*)^27^. Single-cell RNA-seq (scRNA-seq) data from *Pten*^*pc*−*/*−^ tumor-sorted PMN-MDSCs showed that *Clec4n* and *Clec4e* are highly expressed in PMN-MDSCs compared with other receptors of the same family as well as other cell types (Fig. 3a). These data were validated by RT–qPCR analysis in tumor-sorted PMN-MDSCs from TRAMP-C1 mice (Extended Data Fig. 5a). To determine which CLRs were involved in *N. glabratus*-induced MDSCs activation, *Clec7a*, *Clec4n* and *Clec4e* were silenced by using small interfering RNA (siRNA) in BM-MDSCs (Extended Data Fig. 5b,c). We found that knockdown of Dectin-2 (*siClec4n*) suppressed the phosphorylation of SYK, P38 and ERK in BM-MDSCs treated with *N. glabratus* compared with controls, whereas *siClec7a* and *siClec4e* had no effect on these pathways’ activation (Extended Data Fig. 5d).

We then employed *Clec7a* and *Clec4n* wild-type (WT) and knockout mouse models to further confirm the role of Dectin-1 and Dectin-2 in *N. glabratus* recognition by MDSCs. Treatment with *N. glabratus* on Dectin-1-deficient (*Clec7a*^−/−^) BM-MDSCs induced phosphorylation of SYK, P38 and ERK at levels comparable to those observed in WT BM-MDSCs and did not affect the immunosuppressive activity of these cells against T cells (Fig. 3b,c and Extended Data Fig. 5e). In contrast, stimulation of Dectin-2-deficient (*Clec4n*^−/−^) BM-MDSCs with *N. glabratus* abrogated activation of this signaling pathway (Fig. 3b) and significantly reduced their immunosuppressive capacity compared with Dectin-2 WT BM-MDSCs. This effect was evidenced by decreased expression of *Nos2* and *Arg1* and confirmed by T cell suppression assays (Fig. 3c,d). Treatment with mannans, a key component of fungal cell walls and a selective activator of Dectin-2, in BM-MDSCs induced the phosphorylation of SYK, P38 and ERK and increased the messenger RNA expression of *Nos2* and *Arg1*, mirroring treatment with *N. glabratus* (Fig. 3e,f). These changes were accompanied by enhanced immunosuppressive activity in mannan-treated BM-MDSCs, as evidenced by reduced T cell proliferation in a T cell suppression assay (Fig. 3g). Taken together, these data suggest that *N. glabratus* promotes PMN-MDSC activation via Dectin-2.

To further validate these results in vivo, we reconstituted CTX C57BL6/N mice challenged with TRAMP-C1 tumors with BM precursors from WT (*Wt*) and *Clec4n*^−*/*−^ mice to generate the *Wt*_TRAMP-C1 and *Clec4n*^−*/*−^_TRAMP-C1 mouse models. After transplantation, mice were supplemented with or without *N. glabratus*. On treatment with *N. glabratus*, *Clec4n*^−*/*−^_TRAMP-C1 mice developed smaller tumors compared with *Wt*_TRAMP-C1 mice (Fig. 3h), proving that the receptor is required for the *N. glabratus* tumor-promoting effect. Notably, we found that tumors of *Clec4n*^−*/*−^_TRAMP-C1 mice were less infiltrated by PMN-MDSCs than control mice (Fig. 3i and Extended Data Fig. 5f). Taken together, these results indicate that *N. glabratus* promotes both the recruitment and the immunosuppressive function of PMN-MDSCs through direct engagement of Dectin-2. This interaction activates downstream signaling pathways that enhance the suppressive capacity of PMN-MDSCs within the TME, thereby facilitating tumor immune evasion and cancer progression.

### Dectin-2-mediated activin A production by PMN-MDSCs promotes PCa progression

**Fig. 4:**
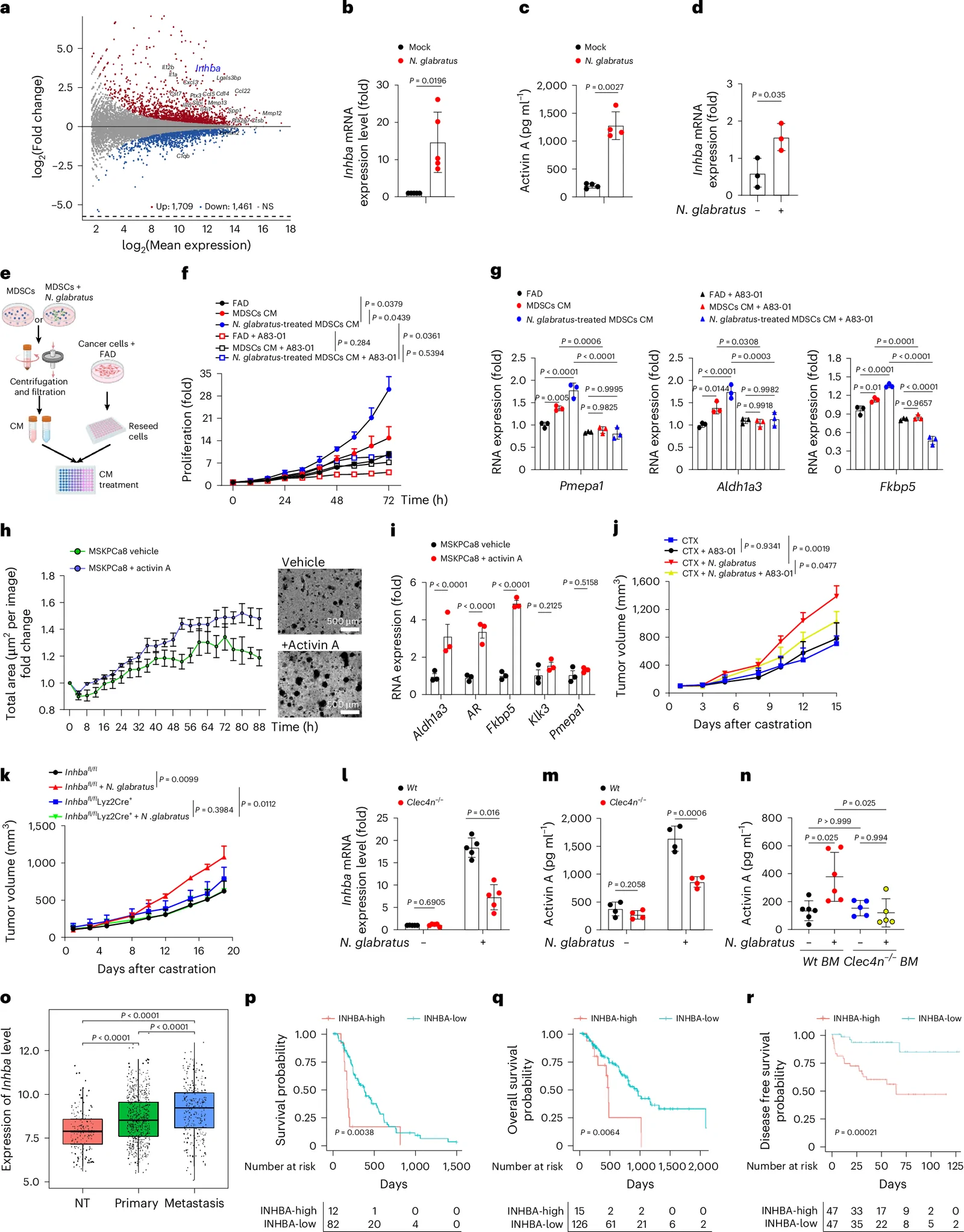
Activin A produced by PMN-MDSCs following *N. glabratus* sensing promotes PCa progression. **a**, RNA-seq analysis of secretome gene expression in BM-MDSCs treated with or without *N. glabratus*. MA plot showing mean expression and log_2_(fold change) of genes encoding for secreted factors. Significantly upregulated genes are represented in red whereas significantly downregulated genes are colored in blue (*n* = 3 biological samples per group). **b**, RT–qPCR analysis of *Inhba* expression in BM-MDSCs treated with or without *N. glabratus*. Each dot represents an independent experiment (*n* = 5 per group). The *P* values were calculated using two-tailed, unpaired Student’s *t*-test with Welch’s correction. **c**, ELISA of activin A level in the supernatant of the BM-MDSC samples. Each dot represents an experiment (*n* = 4 experiments per group). The *P* values were calculated using two-tailed unpaired *t*-test with Welch’s correction. **d**, RT–qPCR analysis of *Inhba* expression in Ly6G^+^Ly6C^int^ CD11b^+^ PMN-MDSCs sorting from TRAMP-C1 tumor-bearing mice treated with or without *N. glabratus*. Each dot represents an individual mouse (*n* = 3 mice per group). The *P* values were calculated using an unpaired, two-sided Student’s *t*-test. **e**, Workflow of FAD TRAMP-C1 cells cultured with BM-MDSC CM (treated with or without *N. glabratus*). **f**, In vitro growth curve of TRAMP-C1 cells cultured with BM-MDSC CM (treated with or without *N. glabratus)* and/or A83-01 (0.5 μM). Data pooled from each dot represent one experiment representing three independent experiments. The *P* values were calculated using two-way ANOVA with Tukey’s multiple-comparison test. **g**, RT–qPCR analysis of androgen-related gene *Pmepa1*, *Aldh1a3* and *Fkbp5* expression in TRAMP-C1 cells cultured with BM-MDSC CM (treated with or without *N. glabratus)* and/or A83-01 (0.5 μM). Each dot represents an independent experiment (*n* = 3 per group). The *P* values were calculated using one-way ANOVA with Tukey’s multiple-comparison test. **h**, Growth curve recorded by incucyte of AR-positive human organoid MSKPCa8 treated with or without activin A (50 ng ml^−1^) for 72 h. Data represent one of three independent experiments. Right: a representative image of organoids treated with vehicle or activin A. **i**, RT–qPCR analysis of the indicated AR-target gene expression in human organoid MSKPCa8 treated as earlier. Each dot represents an independent experiment (*n* = 3 per group). The *P* values were calculated using an unpaired, two-sided Student’s *t*-test. **j**, Tumor size of CTX TRAMP-C1 tumor-bearing mice treated with ABX or ABX + *N. glabratus* and A83-01 (*n* = 4 for each group). The *P* values were calculated using two-way ANOVA with Tukey’s multiple-comparison test. **k**, Tumor size of TRAMP-C1 tumor-bearing *Inhba*^*fl/fl*^ and *Inhba*^*fl/fl*^ Lyz2Cre^+^ mice with CTX and orally given ABX or ABX + *N. glabratus* (*Inhba*^*fl/fl*^, *n* = 6; *Inhba*^*fl/fl*^ + *N. glabratus*, *n* = 4; *Inhba*^*fl/fl*^Lyz2Cre^+^, *n* = 6; and *Inhba*^*fl/fl*^ Lyz2Cre^+^ + *N. glabratus*, *n* = 6). **l**, RT–qPCR analysis of *Inhba* expression in *Wt* and *Clec4n*^−*/*−^ BM-MDSCs treated with or without *N. glabratus*. Each dot represents a value from 1 mouse (*n* = 5 per group). **m**, ELISA of activin A level in the supernatant of *Wt* and *Clec4n*^−*/*−^ BM-MDSCs treated with or without *N. glabratus*. Each dot represents a value from 1 mouse (*n* = 4 per group). The *P* values were calculated using two-sided Mann–Whitney *U*-test with Holm–Šidák multiple comparisons. **n**, ELISA of plasma activin A level in *Wt* and *Clec4n*^−*/*−^ TRAMP-C1-bearing mice treated with or without *N. glabratus*. Data pooled from each dot represent the value from 1 mouse (*Wt* BM, *n* = 6; *Wt* BM + *N. glabratus*, *n* = 6, *Clec4n*^−*/*−^ BM, *n* = 5; and *Clec4n*^−*/*−^ BM + *N. glabratus*, *n* = 5). The *P* values were calculated using an unpaired, two-sided Student’s *t*-test. **o**, Analysis of *INHBA* expression level in nontumor tissue (NT, *n* = 174), primary tumor (*n* = 714) and metastatic tumor (*n* = 316) in the transcriptomic dataset of the Prostate Cancer Atlas. Box plots show the interquartile range (IQR), the horizontal lines the median values and the whiskers extend to the furthest datapoints that are within 1.5× the IQR from the hinges. The *P* values were calculated using one-way ANOVA followed by Tukey’s multiple-comparison test. **p**–**r**, Survival probability of *Inhba*_High and *Inhba*_Low in the ICR-RMH cohort (**p**), SU2C cohort (**q**) and Taylor cohort (**r**). The Kaplan–Meier test has been used in overall survival analysis and the expression cutoff was applied using the Maxstat method. The *P* values were calculated between the high and low groups using log(rank) (Mantel–Cox) tests. All values are presented as the mean ± s.d. Illustration in **e** created in BioRender; Lai, P. https://biorender.com/z69k8vh (2026). Source data

**Extended Data Fig. 6:**
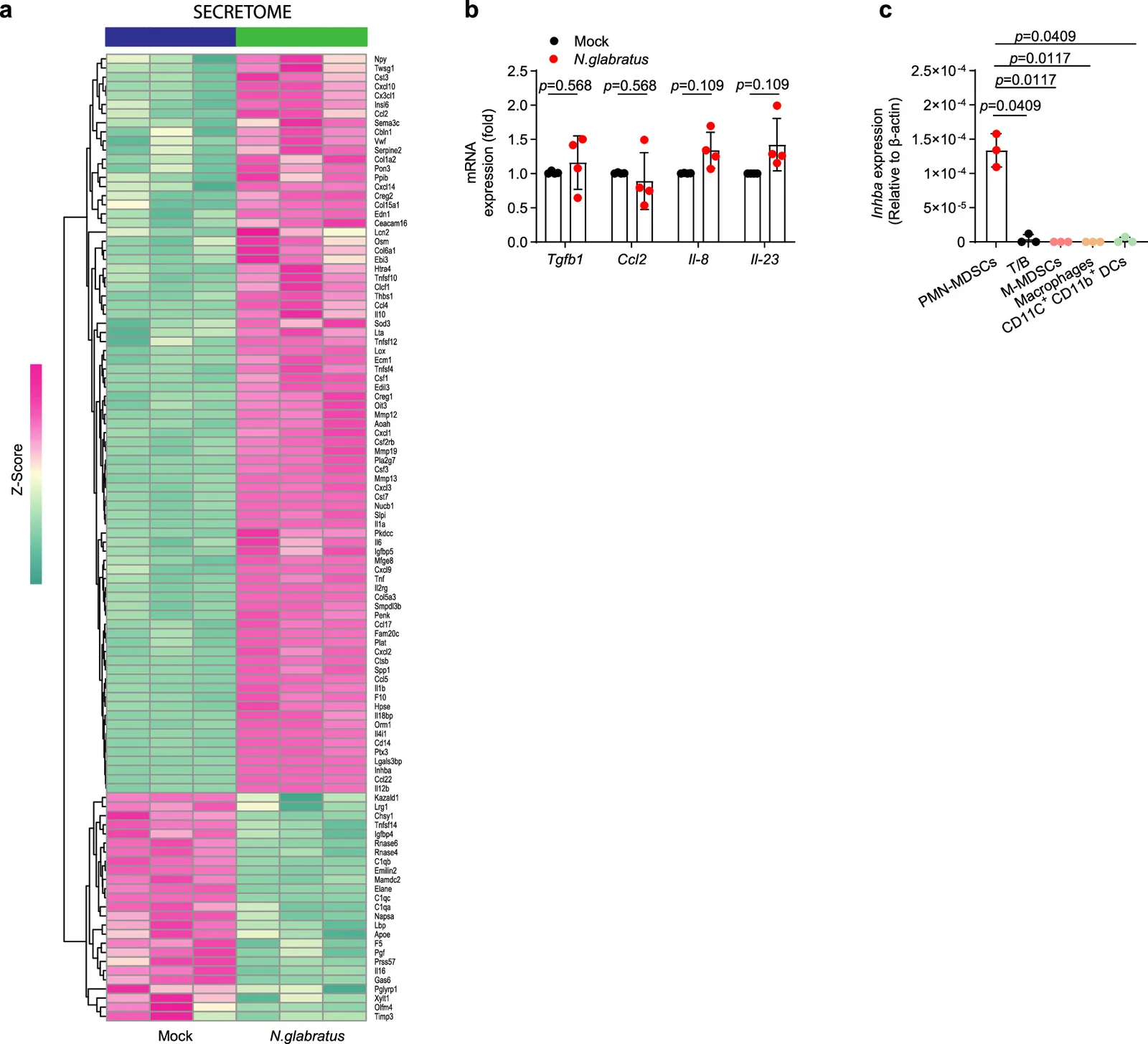
*N.glabratus* drives PMN-MDSCs derived Activin A production. **a**: Heatmap showing the expression of genes encoding for secreted factors in BM-MDSCs treated with or without *N.glabratus* (n = 3 biological samples per group). **b:** RT-qPCR analysis in BM-MDSCs treated with or without *N.glabratus* showing the expression of different genes as indicated. Each dot represents an independent experiment (n = 4 per group). Adjusted *p-*values were calculated using Mann-Whitney U test with Holm-Sidak multiple comparisons. **c:** RT-qPCR analysis of *Inhba* expression in different immune cells sorted from prostate tumor of *Pten*^*pc*−*/*−^ mice (n = 3 per group). Each dot represents the value from one mouse. *p-*values were calculated using Kruskal-Wallis test with FDR multiple comparisons. All values are presented as the mean ± SD. Source data

Previous evidence from other teams and our team demonstrates that PMN-MDSCs promote CRPC by releasing different secreted factors in the prostate TME^19,28^. We therefore assessed which of the factors produced by PMN-MDSCs could promote cancer progression in CTX mice. Bioinformatics analysis of RNA-seq data in BM-MDSCs treated in vitro with *N. glabratus* showed that activin A (encoded by *Inhba*) was one of the most upregulated secreted factors in these cells, whereas the levels of previously identified tumor-promoting cytokines, such as *Il-23*, *Ccl2* and *Il-8*, remained unchanged after *N. glabratus* administration (Fig. 4a and Extended Data Fig. 6a,b). Increased levels of activin A in BM-MDSCs treated with *N. glabratus* were also confirmed by RT–PCR data and ELISA performed in the CM of *N. glabratus*-treated BM-MDSCs (Fig. 4b,c). These findings were validated in vivo by analyzing activin A expression level in tumor-infiltrating PMN-MDSCs and additional immune cell populations sorted from *N. glabratus*-treated mice. Analysis of sorted tumor-infiltrating immune cell populations revealed that *Inhba* expression was highest in PMN-MDSCs compared with macrophages, lymphocytes and monocytes (Extended Data Fig. 6c). Furthermore, *N. glabratus* treatment significantly increased *Inhba* expression in PMN-MDSCs in vivo (Fig. 4d). Activin A is a hormone produced within the gonads, pituitary glands, placenta and other organs, primarily involved in the regulation of reproductive functions^29^. However, recent findings demonstrate that activin A binds to activin receptors expressed on prostate tumor cells, activating SMAD signaling and thereby promoting PCa progression^30^.

**Extended Data Fig. 7:**
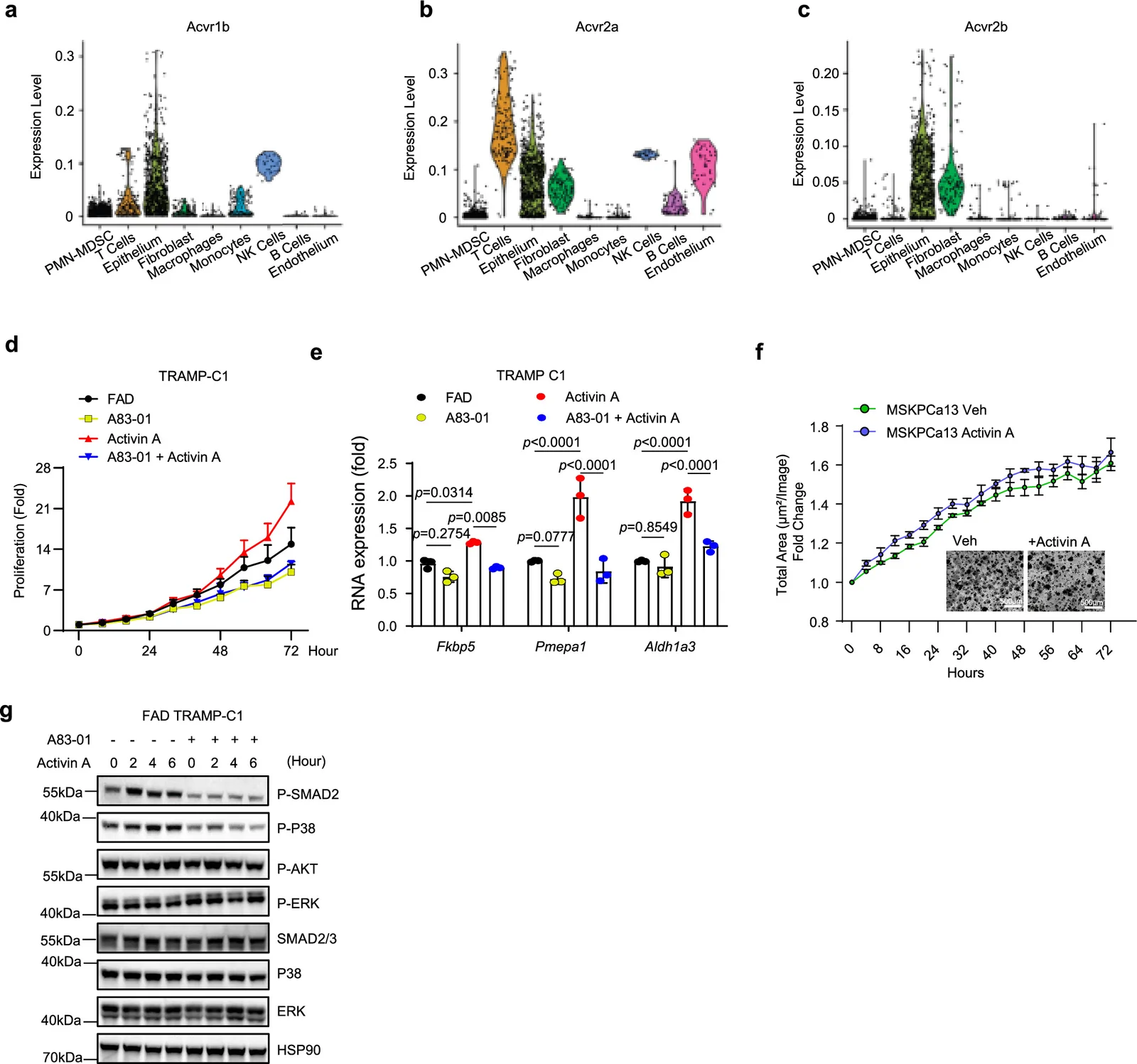
MDSCs-derived Activin A promotes prostate cancer progression via upregulation of AR-related genes. **a-c:** scRNA-seq analysis of prostate tumor in castrated (CTX) *Pten*^*pc*−*/*−^ mouse models. Violin plots show the gene expression of *Acvr1b* (**a**), *Acvr2a*, (**b**) *Acvr2b* (**c**) in different cell types recovered from a prostate tumor. Prostate lobes from two castrated *Pten*^*pc*−*/*−^ mice were pooled equally for single-cell RNA sequencing. **d:** Proliferation curve of TRAMP-C1 cultured in FAD RPMI1640 medium treated with or without Activin A (50 ng/ml) or A83-01 (0.5 µM) at different time points. Data shown are from one experiment representative of three independent experiments. **e:** RT-qPCR analysis of AR-related gene *Pmepa1*, *Aldh1a3*, and *Fkbp5* expression in TRAMP-C1 cells with or without Activin A (50 ng/ml) or A83-01 (0.5 µM). Each dot represents an independent experiment (n = 3 per group). *p-*values were calculated using Two-way ANOVA with Tukey post-hoc test. **f:** Growth curve recorded by IncuCyte of AR-negative human organoid MSKPCa13 treated with Activin A (50 ng/ml) for 72 h. Data shown are representative of one of three independent experiments. **g:** Western blot analysis of signaling pathways in TRAMP-C1 cells cultured in FAD medium treated with or without Activin A (50 ng/ml) or A83-01 (0.5 µM). Representative immunoblots from one of three independent experiments are shown. All values are presented as the mean ± SD. Source data

We then performed scRNA analysis of activin A receptors, including *Acvr1b*, *Acvr2a* and *Acvr2b*, in *Pten*^*pc*−*/*−^ tumors of CTX mice and found that activin A receptors were highly expressed on tumor epithelial cells (Extended Data Fig. 7a–c). TRAMP-C1 prostate tumor cells were in vitro cultured in full androgen deprivation (FAD) conditions in the presence or absence of the CM of BM-MDSCs exposed to *N. glabratus*, plus or minus the activin receptor inhibitor A83-01 (type Ιβ activin receptor) (Fig. 4e). The CM of *N. glabratus-*treated BM-MDSCs promoted tumor cell proliferation (Fig. 4f) and increased the expression of different AR-target genes (Fig. 4g). However, this effect was abolished by the concomitant treatment with A83-01 (Fig. 4f,g). These findings were validated by using activin A recombinant protein in AR^+^ TRAMP-C1 cells in the presence or absence of A83-01 (Extended Data Fig. 7d,e).

Activin A treatment also promoted the proliferation and AR-associated gene expression of the AR^+^ human PCa organoid MSKPCa8, but had no impact on the AR^−^ organoid MSKPCa13 (ref. ^31^; Fig. 4h,i and Extended Data Fig. 7f). Western blotting analysis showed a decreased SMAD phosphorylation in cells treated with A83-01 (Extended Data Fig. 7g). To validate these data in vivo, we treated CTX TRAMP-C1 tumor-bearing mice with *N. glabratus* in the presence of A83-01. Mice treated with A83-01 showed smaller tumors when compared with mice receiving *N. glabratus* alone (Fig. 4j). To further confirm the role of MDSC-derived activin A in *N. glabratus*-induced cancer progression, we generated the *Inhba*^*fl/fl*^*Lyz2Cre*^*+*^ mice in which activin A is specifically depleted in PMN-MDSCs. CTX mice were challenged with TRAMP-C1 tumors and treated with or without *N. glabratus*. We found that *N. glabratus* administration-induced tumor proliferation was decreased in *Inhba*^*fl/fl*^*Lyz2Cre*^*+*^ mice (Fig. 4k).

Finally, we assessed whether *N. glabratus* promotes activin A production in MDSCs by activating Dectin-2 signaling. We found that *N. glabratus* induced activin A production in *Wt* BM-MDSCs, whereas Dectin-2^−/−^ (*Clec4n*^−*/*−^) BM-MDSCs exhibited significantly reduced expression level of *Inhba* and reduced activin A production (Fig. 4l,m). Consistent with the in vitro result, the plasma activin A level in *N. glabratus-*treated *Clec4n*^−*/*−^_TRAMCP-C1 mice was also reduced compared with that of *Wt*_TRAMP-C1-bearing mice (Fig. 4n). Taken together, these data demonstrate that *N. glabratus* activates the Dectin-2-signaling pathway in PMN-MDSCs to promote activin A production, leading to AR-target gene expression and cancer progression. By analyzing publicly available bulk RNA-seq data^32^, we observed a higher expression of *INHBA* mRNA levels in primary and metastatic CRPC tumors compared with normal tissue (Fig. 4o). The increased expression of *INHBA* was also associated with poor survival probability in patients with PCa across different databases (Institute of Cancer Research–Royal Marsden NHS Foundation Trust (ICR-RMH) dataset^33^, the Stand Up to Cancer (SU2C) dataset^34^ and the Taylor dataset^35^) (Fig. 4p–r).

### Gut-derived intratumoral *N. glabratus* promotes cancer progression

**Fig. 5:**
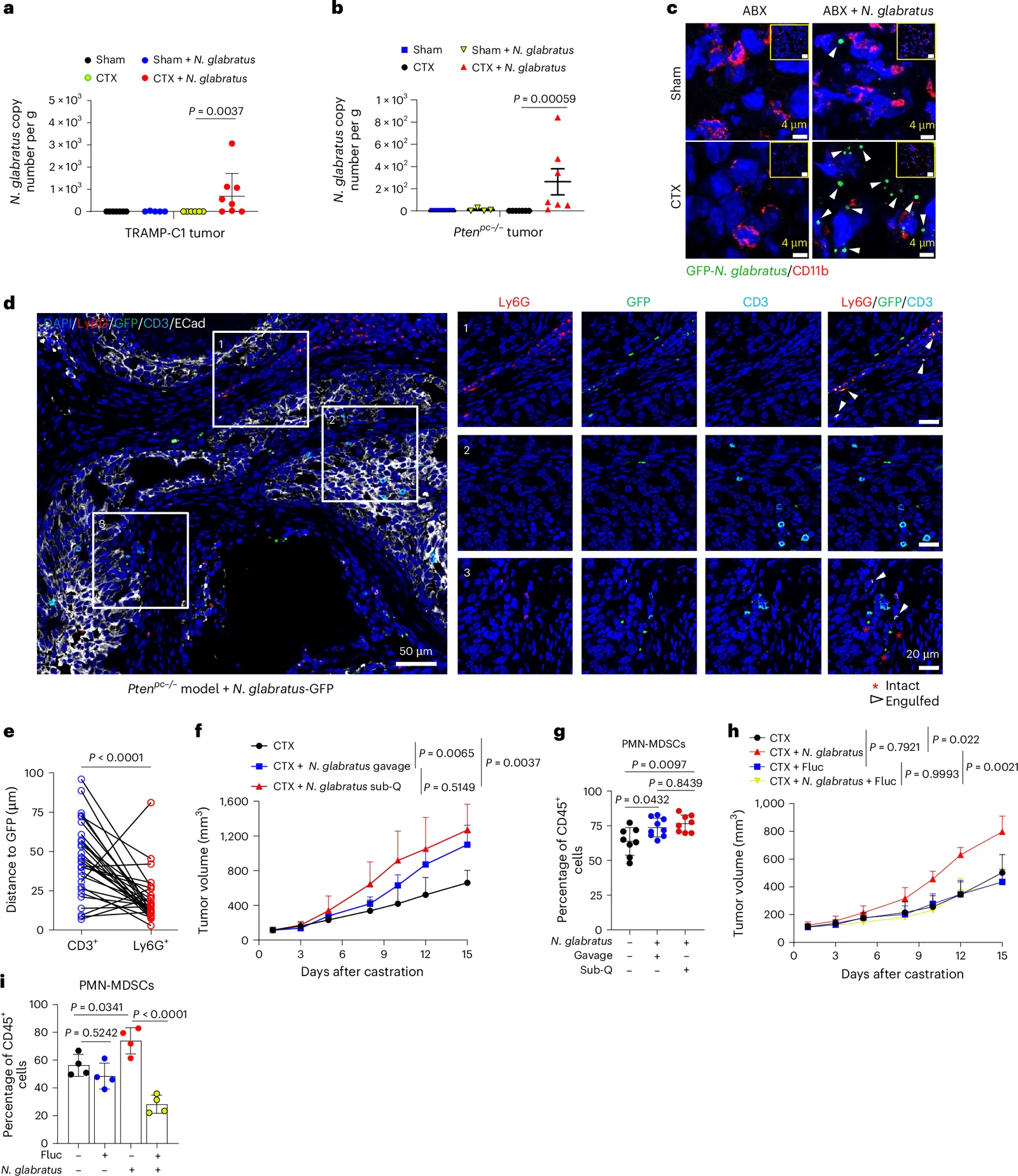
Gut-derived intratumoral *N. glabratus* promotes CTX-resistant PCa progression. **a**, The qPCR analysis of *N. glabratus* copy number in TRAMP-C1 tumor tissues. Each dot represents 1 mouse (sham, *n* = 7; sham + *N. glabratus*, *n* = 5; CTX, *n* = 7; and CTX + *N. glabratus*, *n* = 8). The *P* values were calculated using the Kruskal–Wallis test with Dunn’s multiple comparisons. **b**, The qPCR analysis of *N. glabratus* copy number in the anteroposterior lobes of *Pten*^*pc*−*/*−^ mice. Each dot represents 1 mouse (sham, *n* = 5; sham + *N. glabratus*, *n* = 4; CTX, *n* = 7; and CTX + *N. glabratus*, *n* = 7). The *P* values were calculated using the Kruskal–Wallis test with Dunn’s multiple comparisons. **c**, Confocal microscopy images of indicated TRAMP-C1 tumor tissues (CD11b staining indicates tumor-infiltrating myeloid cells). The white arrows indicate GFP-labeled *N. glabratus* in the tumor. The image shown is from one representative mouse of three analyzed per group. **d**, Confocal microscopy images of anteroposterior lobes of *Pten*^*pc*−*/*−^ mice receiving *N. glabratus* administration as in **b**. The image shown is from one representative mouse of four analyzed per group. The white arrows indicate the engulfed GFP-labeled *N. glabratus* in the tumor and the red stars the intact *N. glabratus*. **e**, The distance of each intact *N. glabratus* to the closest Ly6G^+^ cells and CD3^+^ cells. Each dot represents 1 intact GFP-labeled *N. glabratus* (*n* = 30). The *P* values were calculated using a paired, two-tailed Student’s *t*-test. **f**,**g**, Tumor volume (**f**) and percentage of immune cells (gated on CD45^+^ cells) (**g**) of CTX TRAMP-C1 mice treated with *N. glabratus* orally (gavage) or with sub-Q tumor injection. Each dot represents a value from 1 mouse (CTX, *n* = 8; CTX + *N. glabratus* gavage, *n* = 9; and CTX + *N. glabratus* sub-Q, *n* = 8). The *P* values were calculated using two-way ANOVA with Tukey’s multiple-comparison test in **f** and one-way ANOVA with Šidák’s post-hoc test in **g**. **h**, Tumor volume of CTX TRAMP-C1 mice treated with ABX or ABX + *N. glabratus* and/or subcutaneous tumor injection of Fluc. Data pooled from each dot represent the value from 1 mouse (CTX, *n* = 5; CTX + *N. glabratus*, *n* = 5; CTX + Fluc, *n* = 5; and CTX + *N. glabratus* and Fluc, *n* = 6). The *P* values were calculated using two-way ANOVA with Tukey’s multiple-comparison test. **i**, Percentage of PMN-MDSCs (gated on CD45^+^ cells) of CTX TRAMP-C1 mice treated as mentioned above. Data pooled from each dot represent the value from 1 mouse (*n* = 4 per group). The *P* values were calculated using one-way ANOVA with Šidák’s multiple-comparison test. All values are presented as the mean ± s.d. Source data

**Extended Data Fig. 8:**
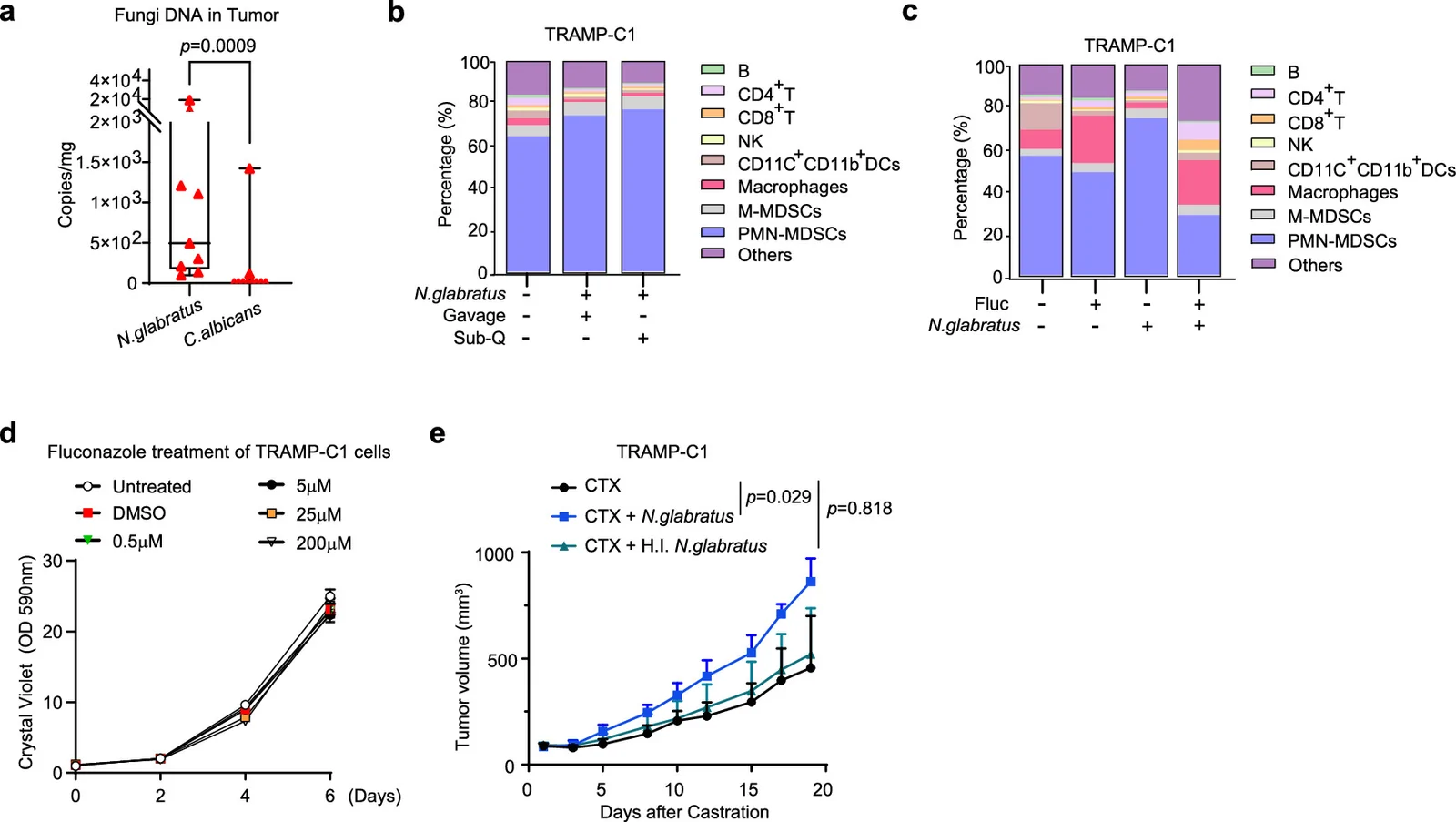
Gut *N.glabratus* migrates to the tumor to promote Castration-resistant prostate cancer progression. **a:** Quantitative PCR (qPCR) analysis of fungal DNA in tumor tissues harvested from TRAMP-C1 bearing mice with oral administered *N.glabratus* or *C.albicans*. Each dot represents the value from one mouse (*N.glabratus*, n = 9; *C.albicans*, n = 10). *p-*values were calculated using unpaired two-sided Student’s t-test. **b:** Percentage of immune cell populations (gated on CD45^+^ cells) of CTX TRAMP-C1 mice treated with *N.glabratus* orally (Gavage) or with subcutaneous (Sub-Q) tumor injection (CTX, n = 8; CTX + *N.glabratus* Gavage, n = 9; CTX + *N.glabratus* Sub-Q, n = 8). **c:** Percentage of immune cell populations (gated on CD45^+^ cells) of CTX TRAMP-C1 mice treated with ABX or ABX plus *N.glabratus* and/or subcutaneous tumor injection of Fluconazole (Fluc) (n = 4 mice per group). **d:** Crystal violet staining of TRAMP-C1 cells treated with fluconazole in indicated concentrations and time points. Data are representative of one of three independent experiments. **e:** Tumor size of TRAMP-C1 mice treated with ABX, *N.glabratus* or heat-inactivated *N.glabratus* (H.I. *N.glabratus*). CTX, n = 5; CTX + *N.glabratus*, n = 5; CTX + H.I *N.glabratus*, n = 5. *p-*values were calculated using Two-way ANOVA with Tukey’s multiple comparisons test. All values are presented as the mean ± SD. Source data

We next hypothesized that, in CTX prostate tumor-bearing mice, intestinal *N. glabratus* may translocate to the tumor site, to directly activate PMN-MDSCs within the TME. To verify this hypothesis, we orally administered GFP-labeled *N. glabratus* to TRAMP-C1 and *Pten*^*pc*−*/*−^ tumor-bearing mice. The qPCR analysis in prostate tumor lysates using *N. glabratus-*specific primers showed that *N. glabratus* was detected at the tumor site only in CTX but not in sham-operated mice (Fig. 5a,b). In contrast, oral administration of *C. albicans* to TRAMP-C1 tumor-bearing mice generally failed to demonstrate intratumoral colonization by this fungus (Extended Data Fig. 8a). Immunofluorescence (IF) staining further revealed the presence of intratumoral GFP-labeled *N. glabratus* in close proximity to CD11b^+^ myeloid cells within prostate tumors from TRAMP-C1 tumor-bearing mice (Fig. 5c). IF staining for Ly6G, CD3 in *Pten*^*pc*−*/*−^ tumors also revealed a clearly co-localization of GFP-*N. glabratus* with Ly6G^+^ cells, but not with CD3^+^ cells (Fig. 5d). Moreover, *N. glabratus* was found in significantly closer proximity to Ly6G^+^ cells (19.03 ± 15.33 µm) compared with CD3^+^ cells (44.73 ± 23.02 µm) (Fig. 5e), indicating the preferential interactions with PMN-MDSCs.

To assess whether the tumor-promoting effect of *N. glabratus* administration required its intratumoral localization, we directly subcutaneously injected *N. glabratus* into the tumor site of TRAMP-C1-bearing mice. Notably, subcutaneous injection (sub-Q) of *N. glabratus* increased tumor growth formation and intratumoral infiltration of PMN-MDSCs, phenocopying the effect of *N. glabratus* oral administration (Fig. 5f,g and Extended Data Fig. 8b). In addition, the deleterious effect of *N. glabratus* oral administration in CTX TRAMP-C1-bearing mice was reversed by the sequential oral administration of an antimycotic treatment, fluconazole (Fluc), which also decreased PMN-MDSC infiltration (Fig. 5h,i and Extended Data Fig. 8c). Notably, Fluc did not directly impact tumor cell proliferation in vitro in TRAMP-C1 cells (Extended Data Fig. 8d). Similarly, oral administration of heat-inactivated *N. glabratus* failed to reproduce the pro-tumor effect observed with live *N. glabratus* administration (Extended Data Fig. 8e). Together, these data suggest that, in CTX prostate tumor-bearing mice, commensal *N. glabratus* migrates from the gut to prostate tumors and that the presence of *N. glabratus* in the tumors promotes cancer progression through enhanced infiltration and activation of PMN-MDSCs.

### Androgen maintains intestinal barrier homeostasis

**Fig. 6:**
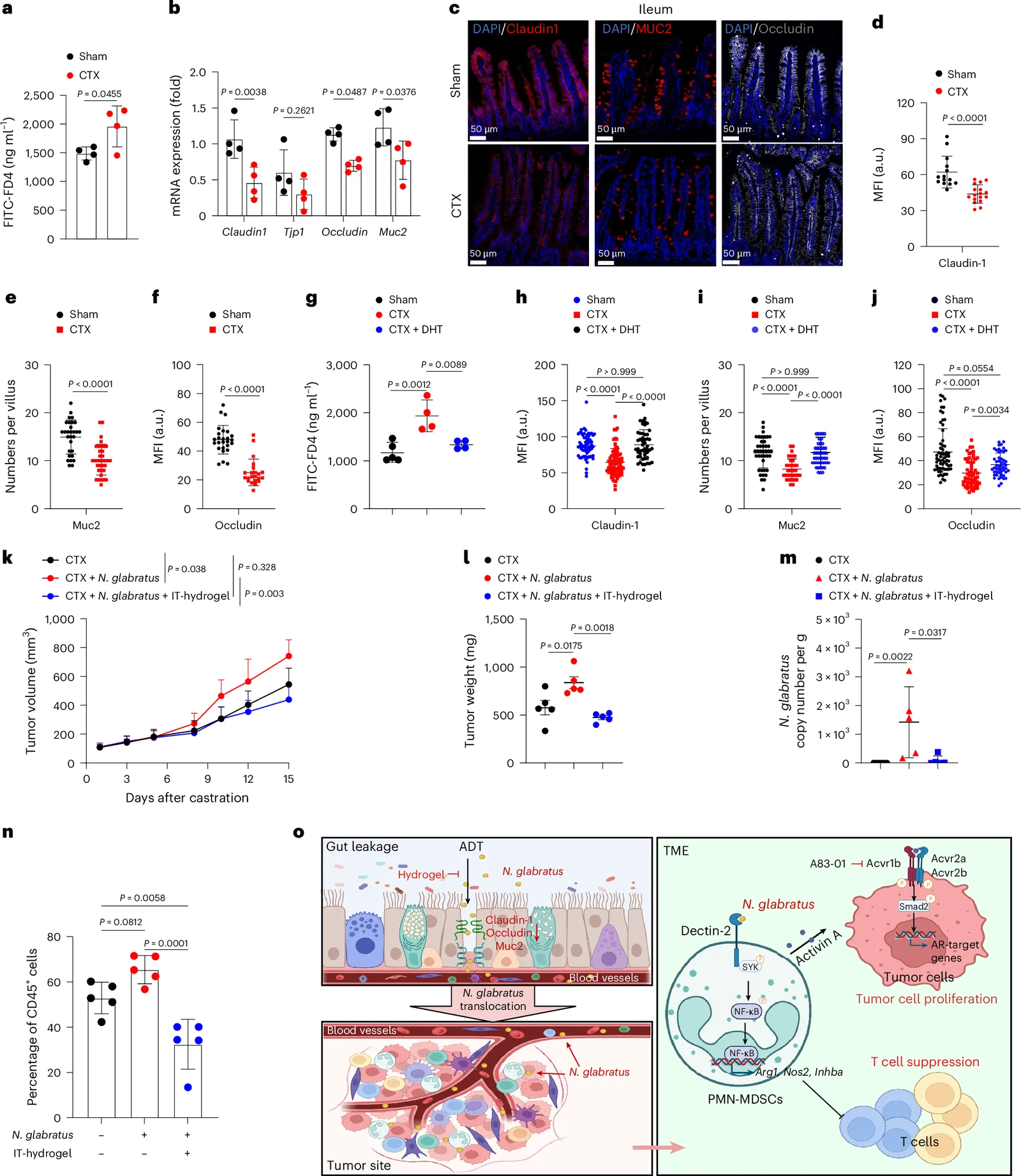
Androgen maintains intestinal barrier homeostasis. **a**, In vivo intestinal permeability assay in sham and CTX mice conducted after 4 h of treatment with FITC–dextran. The FITC concentration was measured using plasma and fluorescence was measured using a spectrofluorometer. Each dot represents a value from 1 mouse (*n* = 4 per group). The *P* values were calculated using an unpaired, two-sided Student’s *t*-test. **b**, RT–qPCR analysis of *Claudin1*, *Tjp1*, *Occludin* and *Muc2* expression in the ileum of sham and CTX mice. Each dot represents 1 mouse (*n* = 4 per group). The *P* values were calculated using two-way ANOVA with Šídák’s post-hoc test. **c**, Representative confocal microscopy images showing Claudin-1 and occludin expression in sham and CTX mice. **d**–**f**, Quantification of mean fluorescence intensity (MFI) of Claudin-1 (**d**), Muc2 (**e**) and occludin (**f**). Each dot represents the MFI value of the indicated protein in each intestinal villus from 3 mice per group (Claudin-1: sham, *n* = 14; and CTX, *n* = 17; Muc2: sham, *n* = 29; and CTX, *n* = 35; occludin: sham, *n* = 26; and CTX, *n* = 22). The *P* values were calculated using a two-tailed Mann–Whitney *U*-test in **d** and **f** or an unpaired, two-sided Student’s *t*-test in **e**. **g**, The FITC–dextran concentration was measured using plasma from sham, CTX and CTX-treated-with-DHT mice. Each dot represents 1 mouse (sham, *n* = 5; CTX, *n* = 4; and CTX + DHT, *n* = 4). The *P* values were calculated using a one-way ANOVA with Tukey’s post-hoc test. **h**–**j**, Mean Claudin-1 (**h**), Muc2 (**i**) and occludin (**j**) intensity in sham, CTX and CTX-treated-with-DHT mice. Each dot represents the MFI value of the indicated protein in each intestinal villus from 3 mice per group (Claudin-1: sham, *n* = 63; CTX, *n* = 75; and CTX + DHT, *n* = 50; Muc2: sham, *n* = 40; CTX, *n* = 37; and CTX + DHT, *n* = 47; occludin: sham, *n* = 63; CTX, *n* = 59; and CTX + DHT, *n* = 50). The *P* values were calculated using the Kruskal–Wallis test with Dunn’s multiple comparisons in **h**–**j**. **k**,**l**, Tumor size (**k**) and tumor weight (**l**) of TRAMP-C1 mice treated with ABX, *N. glabratus* or *N. glabratus* + IT-hydrogel. Each dot represents 1 mouse (CTX, *n* = 5; CTX *N. glabratus*, *n* = 5; and CTX *N. glabratus* + IT-hydrogel, *n* = 5). The *P* values were calculated using a one-way ANOVA with Tukey’s multiple-comparison test. **m**, RT–qPCR analysis of *N. glabratus* in TRAMP-C1 tumor tissues. Each dot represents 1 mouse (CTX, *n* = 5; CTX *N. glabratus*, *n* = 5; and CTX *N. glabratus* + IT-hydrogel, *n* = 5). The *P* values were calculated using a two-tailed Mann–Whitney *U*-test. **n**, Percentage of tumor-infiltrating PMN-MDSCs gated on CD45^+^ cells. Data pooled from each dot represents the value from 1 mouse (CTX, *n* = 5; CTX *N. glabratus*, *n* = 5; and CTX *N. glabratus* + IT-hydrogel, *n* = 5). The *P* values were calculated using one-way ANOVA with Tukey’s multiple-comparison test. **o**, Schematic diagram of gut *N. glabratus* migrates to the TME under ADT and activates PMN-MDSCs to promote PCa progression. All values are presented as the mean ± s.d. Illustration in **o** created in BioRender; Liu, L. https://biorender.com/sihysg5 (2026). Source data

**Extended Data Fig. 9:**
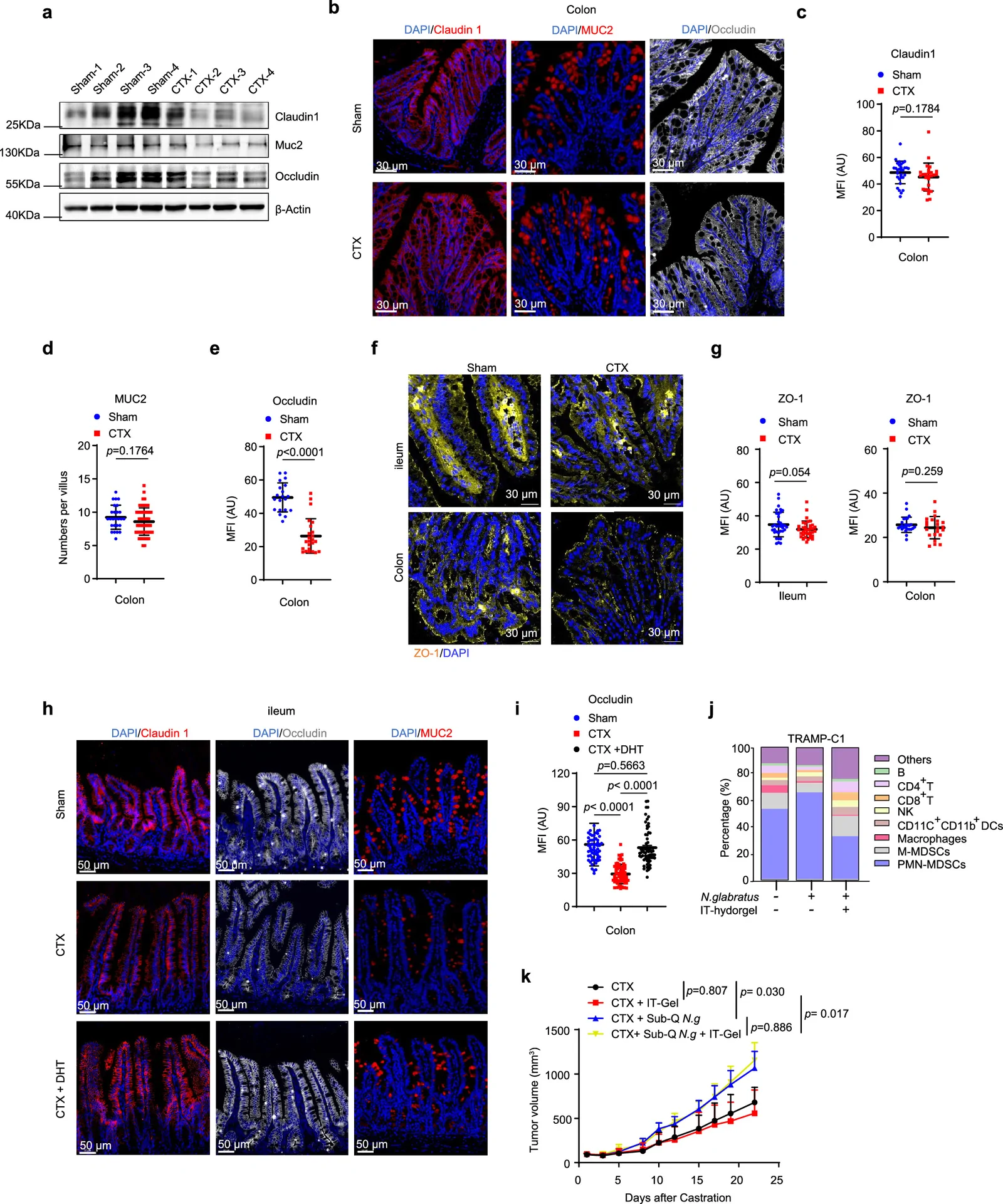
Androgen has less impact on colonic permeability. **a:** Western blot analysis of Claudin1, Muc2 and Occludin in the intestine of sham and CTX mice. **b:** Confocal microscopy images showing Claudin 1, Muc2 and Occludin expression (Claudin1 and Muc2- red, Occludin – white, nucleus- blue) in the colon from sham and CTX mice. Scale bar: 30 μm. **c-e:** Mean intensity of Claudin1 (**c**), Muc2 (**d**), and Occludin (**e**) in the colon villi. Each dot represents the MFI value of the indicated protein in each colon villus from three mice per group (Claudin1: sham, n = 31; CTX, n = 26; Muc2: sham, n = 26; CTX, n = 56; Occludin: sham, n = 20; CTX, n = 24). *p-*values were calculated using unpaired two-sided Student’s t-test. **f, g:** Representative confocal microscopy images (**f**) and MFI quantification (**g**) of ZO-1 expression in sham and CTX mice. Scale bar: 30 μm. Each dot in (**g**) represents the MFI value of the ZO-1 in each ileum or colon villi from three mice per group (left: sham, n = 35; CTX, n = 35; right: sham, n = 25; CTX, n = 22). *p-*values were calculated using unpaired two-sided Student’s t test in (g). **h:** Representative confocal microscopy images showing Claudin 1, Muc2, and Occludin expression in sham, CTX, and CTX supplemented with DHT mice. Scale bar: 50 μm. **i:** MFI of Occludin in the colon tissue from sham, CTX, and CTX supplemented with DHT mice. Each dot represents the MFI value of Occludin in each colon villus from three mice per group (sham, n = 62; CTX, n = 64; CTX + DHT, n = 66). *p-*values were calculated using One-way ANOVA with Tukey’s multiple comparisons test. **j:** Percentage of immune cell population of CTX TRAMP-C1-bearing mice treated with *N.glabratus*, or *N.glabratus* plus IT-hydrogel. (CTX, n = 4; CTX + *N.glabratus*, n = 5; CTX + *N.glabratus* + IT-Gel, n = 4). **k:** Tumor size of TRAMP-C1-bearing mice of CTX, CTX plus IT-hydrogel (IT-Gel), CTX plus Sub-Q *N.glabratus (N.g)* with or without IT-Gel. CTX, n = 6; CTX + IT-Gel, n = 5; CTX + Sub-Q *N.g*, n = 5; CTX + Sub-Q *N.g* + IT-Gel, n = *5*. *p-*values were calculated using Two-way ANOVA with Tukey’s multiple comparisons test. All values are presented as the mean ± SD. Source data

Intrigued by these findings, we next assessed the mechanism by which castration promotes the migration of *N. glabratus* to the tumor site. Antitumor therapies such as radiation therapy and chemotherapy are known to increase intestinal permeability, leading to gut microbiota leakage into the circulating system and subsequent tumor localization^36,37^. In addition, the immunosuppressive or hypoxic TME enhances microbial colonization and survival^38^. However, whether ADT increases intestinal permeability is unclear. To investigate whether ADT can alter intestinal permeability in mice, we administered FITC–Dextran 4-kDa (FD-4) by oral gavage in sham-operated and CTX prostate tumor-bearing mice, and measured the levels of this compound in their blood 4 h later. We found that intestinal permeability was significantly increased in CTX mice compared with sham-operated mice, as evidenced by increased FD-4 levels in the plasma (Fig. 6a). In line with this result, RT–qPCR for different tight junction protein-encoding genes, including *Occludin*, *Claudin-1* and *Muc2*, showed a strong downregulation of these genes in the ileum of CTX compared with sham mice, but not *Tjp-1* (encodes ZO-1) (Fig. 6b). These results were further confirmed by IF staining of the mouse ileum and western blotting analysis of ileum samples from sham and CTX mice (Fig. 6c–f and Extended Data Fig. 9a). The colon from CTX mice showed a decrease in occludin protein levels, whereas Claudin-1, ZO-1 and Muc2 levels remained unchanged (Extended Data Fig. 9b–g). Furthermore, treatment with dihydrotestosterone (DHT) decreased the FD-4 levels in the plasma (Fig. 6g) of CTX mice and restored the expression levels of Claudin-1, occludin and Muc2 in the ileum, as well as occludin in the colon of CTX mice (Fig. 6h–j and Extended Data Fig. 9h,i). Together, these data indicate that androgen regulates the intestinal barrier permeability.

We next assessed whether restoring the intestinal barrier homeostasis in CTX prostate tumor-bearing mice could block the intratumoral localization of *N. glabratus*, thereby inhibiting PCa progression. To test this hypothesis, CTX TRAMP-C1 mice supplemented with *N. glabratus* were treated with an inflammation-targeting hydrogel (IT-hydrogel) that preferentially adheres to inflamed intestinal mucosa. Remarkably, IT-hydrogel treatment markedly suppressed *N. glabratus-*induced tumor proliferation in CTX mice, decreasing the intratumoral migration of *N. glabratus* from the gut (Fig. 6k–m). Consistent with our previous findings, IT-hydrogel treatment also decreased the accumulation of PMN-MDSCs in the tumor site (Fig. 6n and Extended Data Fig. 9j). In contrast, oral IT-hydrogel could not attenuate the tumor growth in mice that received sub-Q *N. glabratus* injection (Extended Data Fig. 9k). Collectively, these results indicate that ADT increases the intestinal barrier permeability, resulting in the leakage of gut *N. glabratus* into the bloodstream and tumor, thereby facilitating PCa progression (Fig. 6o).

## Discussion

Although the continuous presence of microorganisms in the bloodstream of asymptomatic individuals is a debated topic, it is generally accepted that small, transient amounts of microorganisms can occasionally be present without causing symptoms^39^. The immune system plays a crucial role in maintaining this balance by rapidly clearing these microorganisms. After the widespread use of immunosuppressive therapy and broad-spectrum antibiotics, cancer patients are frequently suffering from fungal infections^40^. By analyzing fecal, blood and tumor samples from patients with prostate cancer, we identified *N. glabratus* DNA in an asymptomatic subset of patients with CRPC. Detection of *N. glabratus* DNA in fecal samples was also associated with poorer survival in patients with CRPC. To mitigate the risk of artifacts or environmental noise commonly encountered in conventional computational analyses, we independently validated our findings in metastatic PCa samples using PCR with three specific primer sets, followed by gel electrophoresis and Sanger sequencing. This analysis confirmed that the amplicons originated from clinical *N. glabratus* rather than from laboratory strains. Together, these data substantially reduce the likelihood of false-positive detection and support the genuine intratumoral presence of *N. glabratus* in PCa samples. Although *N. glabratus* was detected in a consistent number of patients’ tumor tissues, the limited sample size precluded a definitive assessment of its association with patients’ disease progression or overall survival. Further studies in larger patient cohorts will therefore be required to validate and extend these preliminary observations.

Experimentally, our mouse models suggest that *N. glabratus* functions as a disease-modifying microbe in primary PCa by promoting tumor progression through PMN-MDSC activation. However, we detected *N. glabratus* in fecal samples from patients with metastatic CRPC. Thus, analyzing fecal samples from patients with primary tumors will help reconcile the apparent discrepancy between our mouse models and the clinical dataset, while strengthening the translational relevance of this study. Mechanistically, we show that ADT can alter the gut vascular barrier permeability in prostate tumor-bearing mice, allowing *N. glabratus* to translocate from the intestine to the bloodstream and tumor site. Previous studies from different teams, including ours, showed that ADT promotes the expansion of intestinal bacteria, which can produce androgens, leading to CRPC^41–43^. Bacterial depletion through antibiotics could impact CRPC progression^41^. Thus, in our experimental setup, we introduced *N. glabratus* into mice that had been pretreated with broad-spectrum antibiotics to prevent the proliferation of bacteria capable of producing androgens. This precaution aimed to isolate the effects of *N. glabratus* without confounding influences from androgen-producing bacteria. Remarkably, our results revealed that the mechanisms governing the migration of *N. glabratus* were influenced by ADT. This finding suggests a direct relationship between androgen signaling and the translocation of *N. glabratus*, underscoring the intricate interplay between hormonal regulation and fungal dynamics within the gut microbiota.

Through compelling evidence, we demonstrate that *N. glabratus*-induced PMN-MDSC activation is dependent on Dectin-2 for fungal recognition by myeloid cells^44^. The underlying mechanism is likely attributable to the structural characteristics of the *N. glabratus* cell wall: a relatively thin β-glucan layer that is masked by a densely mannoprotein-enriched outer layer, thereby preferentially promoting engagement of the mannose-binding receptor Dectin-2 over the β-glucan receptor Dectin-1^45^. Consistently, our study proved that both *N. glabratus* and mannans, a Dectin-2 agonist, upregulated *Arg1* and *Nos2* expression and enhanced the immunosuppressive activity of PMN-MDSCs in vitro and ex vivo. In line with these findings, recent studies have reported that intratumoral fungi promote the immunosuppressive activity of PMN-MDSCs, revealing an in vivo fungi–myeloid cell–T lymphocyte axis in the TME of oral and colorectal cancers^46,47^. Although the in vitro and ex vivo T cell suppression assays suggest that *N. glabratus* promotes an MDSC-like immunosuppressive phenotype, these experimental systems may not fully reflect the in vivo context; thus, the extent to which these cells contribute to T cell-dependent immunosuppression in vivo remains to be determined. Intriguingly, in our study, intratumoral PMN-MDSCs from mice supplemented with *N. glabratus* upregulated activin A without regulating previously described cytokines capable of promoting tumor growth, such as interleukin (IL)-23, CCL2 or IL-8^19,28^. In prostate cancer, activin A has been implicated in the regulation of AR signaling^30^. Activin A can stimulate the transcriptional activity of AR and promote the expression of androgen-responsive genes. Intriguingly, a recent study demonstrated that tumor-associated macrophages can produce activin A in the PCa TME^48^. However, in our study, we found that *N. glabratus* induces activin A production specifically in PMN-MDSCs. Although the *Lyz2-Cre* mouse model lacks strict cellular specificity^49^, our scRNA-seq and in vitro co-culture data indicate that the fungal recognition receptors *Clec4n*, *Clec4e* and *Clec4d* are predominantly expressed in PMN-MDSCs.

In terms of therapeutic implications, we investigated the antitumor effect of an IT-hydrogel previously utilized for treating patients affected by inflammatory bowel diseases^50^. This hydrogel demonstrated a remarkable capacity to prevent the leakage of gut-derived *N. glabratus* into the systemic circulation and its consequent localization in the tumors. By effectively containing the spread of *N. glabratus*, the IT-hydrogel exerted a significant suppressive effect on tumor growth. This antitumor activity was associated with the reduction of tumor-infiltrating PMN-MDSCs, thereby highlighting the potential of this IT-hydrogel as an immunotherapeutic agent capable of targeting the immunosuppressive microenvironment orchestrated by PMN-MDSCs. Furthermore, given the promising clinical outcomes reported in a recent clinical study using inhibitors of MDSCs in patients with metastatic CRPC^28^, the combination of IT-hydrogel with MDSC inhibitors holds considerable promise as a potential synergistic therapeutic strategy. Further studies are required to confirm the antitumor efficacy of this IT-hydrogel strategy in multiple mouse models before evaluating its safety and efficacy in human clinical trials. In conclusion, implementation of this therapy in clinical settings could offer a potent and versatile treatment option for patients with PCa, addressing both fungal-driven mechanisms of tumor progression and immunosuppressive barriers to effective anti-cancer immune responses.

## Methods

All animal experiments were approved by the local ethical committee (Dipartimento della sanità e della socialità, Esperimenti su animali, Canton Ticino, authorization nos 34303 and 37779). Blood from healthy donors was obtained from the Swiss Blood Donation Center of Basel and Lugano and used in compliance with the Federal Office of Public Health (authorization number A000197/2). All patients with PCa provided written informed consent and were enrolled in institutional protocols approved by the RMH (London, UK) ethics review committee (reference number 04/Q0801/60) and within an Ente Ospedaliero Cantonale (EOC)-specific protocol (nos CE TI 3269, IOSI-IOR-002 and CE TI 3831, IOR-FLO-001) and Padua Province Clinical Experimentation Ethics Committee (reference number CESC 5170/AO/21). Demographic and clinical data for each patient were collected from the hospital electronic patient record (Supplementary Tables 1–4).

### Mice

The *Clec4n*^−*/*−^ (C57BL/6J background) mice were obtained from Y. Iwakura (University of Tokyo). The *Clec7a*^−*/*−^ mice, originally constructed by G. Brown (University of Cape Town), were obtained from S. LeibundGut-Landmann (University of Zurich). *Inhba*^*flox/flox*^ mice were generated by M. M. Matzuk (Baylor College of Medicine) and then backcrossed to the C57BL/6N background by S.-j. Lee from the Jackson Laboratory. *Lyz2-Cre* mice were purchased from the Jackson Laboratory (strain number 004781). All mice were propagated under specific pathogen-free or biosafety level 2 conditions, maintained at a constant temperature of 22 ± 2 °C with 50–60% relative humidity and a 12-h light-to-dark cycle and provided with free access to standard laboratory chow and sterile drinking water.

### In vivo experiments

C57BL6/N mice were challenged with 2.5 × 10^6^ of TRAMP-C1 cells and castrated when tumors were approximately 100 mm^3^. For microbiota depletion, mice received a cocktail of neomycin (1 g l^*−*1^), ampicillin (1 g l^*−*1^) and vancomycin (0.5 g l^*−*1^) in drinking water, together with oral metronidazole administration (2 mg, 3× a week). Tumor growth and survival were monitored until tumors reached ~1,500 mm^3^. Male *Pten*^*pc−/−*^ mice were generated and genotyped as previously described and surgically castrated at age 8–10 weeks under anesthesia with isoflurane^19,51^. In fungal administration experiments, the tumor-bearing mice were orally administered with 100 μl of 10^8^ colony-forming units (c.f.u.) of *N. glabratus* after castration. In addition to the maximum tumor volume limit (1,500 mm^3^), several predefined humane endpoints were strictly enforced, including ≥15% body weight loss, reduced mobility, signs of distress (including apathy, social isolation, persistent hunching, aggression on handling, eyes more than half closed, nasal bulging, backward or outward ear rotation and severe piloerection), diarrhea and tumor ulceration.

For hydrogel experiments, IT-hydrogel (4% w:v) was prepared using dimethyl sulfoxide (DMSO) and H_2_O as a solvent (ratio of DMSO to H_2_O = 1:4). After dissolving ascorbyl palmitate in DMSO, sterile H_2_O was added dropwise. The suspension was then heated to 70 °C for 20–30 min and cooled down to room temperature. The formed IT-hydrogel was washed 3× with phosphate-buffered saline (PBS) and centrifuged at 5,000*g* for 10 min. IT-hydrogel was resuspended in 1 ml of sterile PBS; mice were orally administered with 100 µl of IT-hydrogel or 100 µl of PBS as a control.

For PMN-MDSC depletion in vivo, mice were intraperitoneally administered 150 µg of anti-Ly6G antibody (clone 1A8, Bio X Cell, catalog number BP0075-1) 3× weekly until the experiment endpoint. An InVivoPlus rat IgG2a isotype control (clone 2A3, Bio X Cell, catalog number BP0089) was used as a control. For immunodetection of PMN-MDSCs in tumor tissues, we subsequently used an anti-mouse Ly6G antibody (clone S19018G, BioLegend, catalog number 164504) to assess PMN-MDSC infiltration in the TME.

### Cell lines

TRAMP-C1 (catalog number CRL-2730) and LNCaP (catalog number CRL-1740) cell lines were purchased from American Type Culture Collection (ATCC) and B6 Myc-CaP was a kind gift from J.-P. Theurillat (Institute of Oncology Research, Switzerland). PBMCs were isolated from buffy coats obtained from healthy male donors through the Swiss Blood Donation Center in Basel and Lugano. All cell lines were kept under controlled temperature (37 °C) and CO_2_ (5%) and tested negative for *Mycoplasma* spp. (MycoAlert Mycoplasma Detection kit, Lonza, catalog number LT07-218).

### Fungi

*Nakaseomyces glabratus* (*Candida glabrata*) (ATCC, catalog number 2001TM) was purchased from ATCC. *C. albicans* (DSM, catalog number 11943) was purchased from Leibniz Institute DSMZ—German Collection of Microorganisms and Cell Cultures GmbH. Azole-susceptible *N. glabratus* (strain DSY562) and GFP-labeled *N. glabratus* (strain SFY168) were gifted from D. Sanglard (Lausanne University Hospital, Switzerland). All fungi were cultured in yeast mold (YM) broth or agar at 37 °C overnight. For the preparation of heat-inactivated fungi, the cultures were inoculated for 30 min at 60 °C and washed with PBS 3×. The resultant pellet was sampled and inoculated on to YM agar medium to verify the inactivation efficiency.

### Collection of patient samples

Fecal samples were collected using FecalSwab (Copan) and immediately stored at −80 °C. The fresh-frozen tumor samples were collected in a sterile environment using sterile devices in the operating room with a sterile endobag and the sampling was performed immediately and stored at −80 °C in a sterile tube. The remaining tissue samples used were conserved at −80 °C in the corresponding author’s laboratory.

### DNA extraction and whole-metagenome sequencing of patient feces

Whole-metagenome analysis was performed on patients’ feces. DNA was isolated with QIAamp Fast DNA Stool Mini Kit (QIAGEN, catalog number 51604) with the addition of a bead-beating step: 180–220 mg of stool samples was added to 1 ml of 70 °C prewarmed InhibitEX buffer (QIAGEN, catalog number 19593) containing 600–700 mg of 0.1-mm glass beads. The suspension was heated for 5 min at 95 °C and shaken at 1,000–1,200 rpm. The manufacturer’s protocol was followed to isolate the DNA. The extracted DNA was analyzed by Qubit (Invitrogen) and integrity was checked using agarose gel electrophoresis.

For the whole-genome sequencing, Illumina TruSeq DNA libraries were prepared using the TruSeq nano DNA Library kit (Illumina) according to the manufacturer’s instructions. Subsequently, libraries were checked on a 2100 Bioanalyzer instrument, quantified and equimolarly pooled before sequencing. Then, the libraries were sequenced on the Illumina NovaSeq 6000 platform. Paired-end reads that passed Illumina’s chastity filter were demultiplexed and trimmed of Illumina adapter residuals using bcl2fastq software (v2.20). Sequencing reads were filtered by Trimmomatic v0.39 using the following parameters: MINLEN:70 AVGQUAL:30. Alignment to the human reference genome (GRCh38) was performed with Bowtie2 v2.5.1 to remove host-derived sequences. High-quality and clean reads were analyzed with MetaPhlAn v4.0.6 and the reference database mpa_vOct22_CHOCOPhlAnSGB_202212 to profile the composition of the bacterial communities. Fungal species were analyzed using Kraken2 v2.1.3 and the publicly available database kraken2_PlusPF_DB, setting the confidence level at 0.2.

For ITS sequencing, Nextera-barcoded PCR libraries were created using the locus-specific primer pairs ITS3 (5ʹ-TCGTCGGCAGCGTCAGATGTGTATAAGAGACAGNNNNNGCATCGATGAAGAACGCAGC-3ʹ) and ITS4 (5ʹ-GTCTCGTGGGCTCGGAGATGTGTATAAGAGACAGNNNNNTCCTCCGCTTATTGATATGC-3ʹ) with 20 PCR cycles for the first step and 15 for the second step. Libraries were quantified, pooled equimolarly and sequenced on a NovaSeq 6000 platform. Adaptors were trimmed and the reads were quality filtered or subsampled. Reads were merged using Usearch (Edgar, 2010) and operational taxonomic unit clustering was performed, including denoising, before the taxonomic classification of the operational taxonomic unit sequences against the Ribosomal Database Project (database) using the SINTAX algorithm.

### DNA extraction and 16S RNA gene sequencing

Mouse fecal samples were extracted using the DNeasy PowerSoil Pro kit (QIAGEN)^52^. Briefly, 50-80 mg of each sample of feces was added to a PowerBead ProPlate well, along with three wells of water control. Stool was homogenized by bead beating. After centrifugations, samples were then processed using the DNeasy Powerlyzer-Power soil kit following the manufacturer’s protocol. After the extraction, the 585 bp of 16S RNA V3–V4 hypervariable region was amplified using barcoded primer pairs with universal Illumina paired-end adapter sequences and unique individual nucleotide barcodes for multiplex PCR sequencing. Samples were pooled in a sequencing library and purified by AMPure XP kit (Beckman Coulter). The KAPA library quantification kit was used for quantification of the final library and samples were sequenced on Illumina MiSeq.

### Analysis of 16S RNA gene sequences

The 16S ribosomal RNA gene-sequencing data were processed using the DADA2 pipeline^53^ primer trimming, quality filtering, error correction, read merging, amplicon sequence variant (ASV) inference, chimera removal and taxonomic assignment, based on the SILVA database (v138.1). Nonbacterial ASVs were excluded from downstream analyses. Data analyses were done as described previously^41^. Briefly, principal coordinate analysis was generated using the R phyloseq package^54^ from Bray–Curtis dissimilarity on log(transformed counts) with the phyloseq R package^55^. Alpha diversity (Chao1 index) was calculated after rarefaction to the minimum sequencing depth. Statistical analyses accounted for cage effects using the nlme package^55^ and false discovery rates were controlled using the Benjamini–Hochberg method^56^. For taxa analyses at the genus level, ASVs were aggregated according to their taxonomic classification.

### MDSC generation and MDSC CM collection in vitro

Mouse BM-MDSCs and BMDMs were differentiated in vitro from BM cells with Roswell Park Memorial Institute (RPMI)-1640 medium^57,58^. In brief, BM was lysed with ACK buffer (Gibco, catalog number A1049201) and differentiated in RPMI containing 40 ng ml^−1^ of granulocyte colony-stimulating factor (G-CSF,) 40 ng ml of IL-6, 10% fetal bovine serum (FBS) and 1% penicillin–streptomycin for 4 days. For CM collection, BM-MDSCs were cultured in fresh medium containing charcoal-stripped FBS and stimulated with heat-inactivated *N. glabratus* (MOI = 5) for 12 h. CM was collected by sequential centrifugation and filtration (0.2 μm) to remove cells, debris and fungal particles.

Human MDSCs were differentiated in vitro as previously described^59^. In brief, the health donor peripheral blood mononuclear cells (PBMCs) were separated by Ficoll (Sigma-Aldrich) density gradient. Myeloid cells were isolated from PBMCs using anti-CD33 magnetic microbeads and LS column separation (Miltenyi Biotec), differentiated in RPMI-1640 medium containing human granulocyte–macrophage-CSF (10 ng ml^−1^) and human IL-6 (10 ng ml^−1^).

### Extraction of total DNA from frozen patients’ tissue samples

Total DNA was extracted from up to 25 mg of frozen patient tumor tissues using the DNeasy Blood & Tissue Kit (QIAGEN) according to the manufacturer’s protocol, with an additional enzymatic digestion. Briefly, enzymatic digestion was performed sequentially with lyticase (20 U per 100 µl, 45 min, 37 °C) and lysozyme (15 mg ml^−1^, 45 min, 37 °C) to ensure complete cell wall disruption. The manufacturer’s protocol was then followed to isolate the DNA. All the steps were performed under sterile conditions. An empty sterile tube was used and went through all the reagents and steps as the extraction blank control.

The extracted DNA was used for touchdown PCR amplification by using total fungi, *Candida* spp. and *N. glabratus*-specific primers (Supplementary Table 5); 0.1% diethylpyrocarbonate (DPEC)-treated water and GFP-labeled *N. glabratus* served as negative and positive controls, respectively. The PCR products were checked by agar gel electrophoresis. The *N. glabratus* amplicons with the correct size were inserted into a pMD19-T Easy vector (TaKaRa, catalog number 3271) for Sanger sequencing.

### Extraction and quantification of plasma cell-free DNA

The cell-free DNA was extracted from 200 µl of human plasma using MagMAX Cell-Free DNA Isolation Kit (Thermo Fisher Scientific, catalog number A29319) following the manufacturer’s instructions. To minimize the risk of contamination, samples were collected using strict standard operating procedures and all the analytical operations were performed in sterile conditions, adding multiple negative controls (DEPC-treated water). Quantitative PCR was performed for total fungi, *Candida* spp., *C. albicans* and *N. glabratus* using different primers to calculate the absolute copy numbers. The copy number was calculated using the standard curves for cloned fungi, *Candida* spp., *C. albicans* and *N. glabratus* amplicons in a pMD19-T Easy vector, respectively. The samples detected as positive for all three primers were counted as *N. glabratus-*positive cases.

### Cell stimulation

BM-MDSCs, 10^6^, were plated in 12-well plates and stimulated with heat-inactivated *N. glabratus* (MOI = 5), mannan (5 µg ml^−1^) for different timepoints and collected for western blotting or RNA isolation. TRAMP-C1 or LNCaP cells were cultured in charcoal-stripped FBS medium plus enzalutamide 10 μM for 72 h and seeded in FAD medium. Then, cells were stimulated with MDSC CM (1:3), activin A (50 ng ml^−1^) or Plus A83-01 (1 μM) with different timepoints and collected for RNA isolation. The reagents and antibodies used are listed in Supplementary Table 6.

### Human organoids

The PCa patient-derived organoid (PDO) MSKPCa8 (AR^+^) and MSKPCa13 (AR^−^)^31^ culture largely followed the protocol published in ref. ^60^ using epithelial prostate niche factors: 5 ng ml^−1^ of epidermal growth factor, 100 ng ml^−1^ of Noggin and 100 ng ml^−1^ of R-Spondin. The PDO maintained culture in base medium with additional metabolites and growth factors to improve proliferation and viability. Additional factors included: 2 mM GlutaMAX (Gibco, catalog number 35050-068), 1 mM sodium pyruvate (Gibco, catalog number 11360070) and 2 ng ml^−1^ of human NRG1 recombinant (Peprotech, catalog number 100-03). PDOs were embedded in Matrigel matrix (Corning, catalog number 356231) and seeded in replicates in 96-well format optical plates (Corning, catalog number 3904). For the FAD treatment, the DHT and A83-01 were removed from the PDO base medium. After 3 days of FAD treatment (baseline, time 0), the activin A (50 ng ml^−1^) recombinant was added to the medium. The organoid proliferation assay was assessed by an Incucyte S3 Live-Cell Analysis System (SARTORIUS).

### In vitro T cell suppression assay

The isolation of naive splenocytes was labeled with 2.5 μM carboxyfluorescein succinimidyl ester (CFSE; Thermo Fisher Scientific, catalog number C34554) and then anti-CD3 or anti-CD28 beads (Gibco, catalog number 11452D) were added to activate T cells according to the manufacturer’s instructions. Either untreated or *N. glabratus*-treated BM-MDSCs were co-cultured with splenocytes. For the human T cell suppression assay, the isolation of human PBMCs was labeled with 2.5 μM CFSE and then anti-CD3 or anti-CD28 beads (Gibco, catalog number 11132D) were added to activate T cells according to the manufacturer’s instructions. Either untreated or *N. glabratus*-treated hMDSCs were co-cultured with human PBMCs. After 3 days, the proliferation of CFSE-labeled CD8^+^ T cells and CD4^+^ T cells was analyzed by flow cytometry.

### Adoptive transfers

For BM chimera experiments, 10^7^ viable C57BL/6 or *Clec4n*^−*/*−^ BM cells were intravenously transferred into lethally irradiated C57BL/6N mice (10 Gy). Recipient *Pten*^*pc*−*/*−^ mice were lethally irradiated (10 Gy) and transplanted intravenously 2 h later with 1 × 10^7^ viable BM cells from either C57BL/6 or *Clec4n*^−*/*−^ mice.

### IF and immunohistochemistry

AP lobes from *Pten*^*pc*−*/*−^ mice and TRAMP-C1 tumor tissue samples were fixed in 10% neutral-buffered formalin and washed according to standard protocols. Sections (5 μm) were prepared for antibody detection or hematoxylin and eosin (H&E) staining. Images were scanned with Aperio and opened with ImageScope v12.3.2.8013 (Leica Biosystem). For histopathological characterization of the prostate tumors of *Pten*^*pc*−*/*−^ mice, H&E sections of prostate tumors were assessed for normal-like, prostatic intraepithelial neoplasia, adenocarcinoma and invasive carcinoma. The H&E-stained sections were independently evaluated by a prostate pathologist in a blinded fashion, without knowledge of the experimental groups. For each mouse, one representative prostate section was stained and scored according to predefined histological criteria^61^.

For IF, frozen intestinal sections (8 μm) were fixed with 4% paraformaldehyde, permeabilized and blocked following the standard protocol. The anti-Claudin 1, anti-occludin 1 or anti-MUC2 antibodies were incubated at 4 °C over night and stained with secondary antibodies at room temperature. Slides were covered with mounting medium with DAPI (Invitrogen, catalog number P36931) after washing and were visualized under a fluorescent confocal microscope. Images were acquired on a Leica TCS SP5 and analyzed using ImageJ software (National Institutes of Health (NIH)).

### Bioinformatic analysis of transcriptomes and survival outcomes

The RNA-seq of BM-MDSCs was performed using the NEBNext Ultra Directional II RNA library preparation kit and sequenced on the Illumina NextSeq500 with single-end, 75-bp-long reads. Reads were quality checked with FastQC, aligned to the mouse reference genome (GRCm39) using STAR and quantified with GENCODE vM27 annotations. Genes without counts were removed for the analysis and differential expression analysis was performed using DESeq2. Pathway analysis was performed using fgsea and a customized gene signature of MDSC functions from different works^22–26^. All graphic representations were edited using ggplot2 and pheatmap functions.

The publicly available bulk RNA-seq data from the Prostate Cancer Atlas (https://prostatecanceratlas.org/home)^32^ and CRPC transcriptomes (ICR-RMH, SU2C and Prostate Cancer Foundation and Taylor) were used to assess *INHBA* mRNA levels and its association with patient survival. The Kaplan–Meier curve for disease-free survival in patients with PCa was performed, stratifying the patients based on *INHBA* mRNA expression levels. Patients were stratified in tertiles and the comparison reported the third and the first tertiles.

Survival analysis and multivariate Cox’s proportional hazards regression were performed using the R package survival (v3.6-4) on patients with CRPC stratified by the presence versus absence of *N. glabratus* in the gut metagenome. Overall survival was used as the time-to-event variable, with death defined as the event. HRs and 95% CIs of clinical variables from the multivariate Cox’s regression model were visualized using a Forest plot that was generated. Statistical details are provided in the figure legends.

For scRNA-seq analysis, we used data generated by using PCa of *Pten*^*pc*−*/*−^ prostates from ref. ^59^ and checked the expressions of the genes of interest.

### RT–qPCR

For cells and tissues, total RNA was extracted using TRIzol reagent (Ambion, catalog number 15596026) in accordance with the manufacturer’s instructions and complementary DNA was synthesized using an ImPROM II kit according to the manufacturer’s protocol (Promega, catalog number A3800). RT–PCR was performed using Gotaq qPCR master mix (Promega, catalog number A6002) by a Step One Real Time PCR System (Applied Biosystems). The target genes were normalized to the housekeeping gene (β-actin) shown as 2^−Δ*Ct*^. The used primers are listed in Supplementary Table 5.

### In vivo permeability assay

For in vivo permeability assay, the mice were kept without water and food overnight and then were orally administered water containing FITC–dextran (50 mg kg^−1^; Sigma-Aldrich). After 4 h, the plasma was collected and diluted twofold in PBS and the fluorescence was determined at 528 nm with excitation at 485 nm.

### Statistical analysis

Unless otherwise indicated in the figure legends, all data are expressed as the mean ± s.d. All *n* values used for statistical analyses denote biologically independent samples or experiments. No statistical methods were used to predetermine sample sizes but our sample sizes are similar to those reported in previous publications^4,5,8,18,19,28,41^. Animals were randomly assigned to distinct experimental groups and all experimental procedures were implemented in a randomized manner. The investigators were not blinded to group allocation and experimental conditions during experiments and outcome assessment. Data collection and analysis were not performed blind to the conditions of the experiments. Data collection and statistical analysis were conducted objectively without any potential bias. No datapoints or animals were excluded from the analyses. The GraphPad Prism 10 software was used for the statistical analysis. All data underwent normality distribution analysis and homogeneity of variance testing. For multi-group data that satisfied normality, a two-way ANOVA or one-way ANOVA was used, whereas an unpaired, two-tailed Student’s *t*-test was applied for two groups. For data that violated the assumptions for normality or homogeneity of variance, nonparametric tests were utilized as appropriate. *P* < 0.05 was considered significant.

### Reporting summary

Further information on research design is available in the Nature Portfolio Reporting Summary linked to this article.

## Supplementary information

Supplementary Information Supplementary Fig. 1.

Reporting Summary

Supplementary Tables Supplementary Tables 1–6.

## Source data

Source Data Fig. 1 Statistical source data.

Source Data Fig. 2 Statistical source data.

Source Data Fig. 3 Statistical source data.

Source Data Fig. 4 Statistical source data.

Source Data Fig. 5 Statistical source data.

Source Data Fig. 6 Statistical source data.

Source Data Extended Data Fig. 2 Statistical source data.

Source Data Extended Data Fig. 3 Statistical source data.

Source Data Extended Data Fig. 4 Statistical source data.

Source Data Extended Data Fig. 5 Statistical source data.

Source Data Extended Data Fig. 6 Statistical source data.

Source Data Extended Data Fig. 7 Statistical source data.

Source Data Extended Data Fig. 8 Statistical source data.

Source Data Extended Data Fig. 9 Statistical source data.

Unprocessed gels or blots Unprocessed western blot bands and gels.

## Data Availability

The RNA-seq data of BM-MDSCs used in this study are available in the National Center for Biotechnology Information’s (NCBI’s) Gene Expression Omnibus (GEO) database under accession no. GSE311900. The 16S sequencing data from mouse fecal samples are available in the NCBI Sequence Read Archive (SRA) database under BioProject no. PRJNA1490896. Whole-genome metagenomics data and ITS sequencing data from human fecal swabs generated in this study are available in the NCBI SRA database under BioProject no. PRJNA1371343. The previously publicly available human fecal metagenomics data re-analyzed in this study are available in the NCBI SRA database under BioProject no. PRJNA749645. Previously published scRNA-seq data that were re-analyzed here are available under accession no. GSE272965. Bulk RNA-seq data from the SU2C–Prostate Cancer Foundation cohort were downloaded from https://www.cbioportal.org/study/summary?id=prad_su2c_2019. Bulk RNA-seq data from the Taylor cohort were available in the GEO database under accession no. GSE21034. The previously published ICR-RMH cohort of the transcriptomic data from fresh metastatic CRPC tissue biopsies is available under restricted access in the European Genome–phenome Archive under accession no. EGAS50000001269. Access can be requested via the European Genome–phenome Archive. All remaining data supporting the findings of this study are available within the article and Supplementary Information and/or are available upon request from the corresponding authors. Source data are provided with this paper.
