# Supplementary material for: Commensal Nakaseomyces glabratus migrates into prostate tumors to accelerate cancer progression
Source: Nat Cancer. 2026 Sep 9;7(10):1592–609. doi: 10.1038/s43018-026-01229-9 (PMC13694241; doi:10.1038/s43018-026-01229-9)

Figure 3b

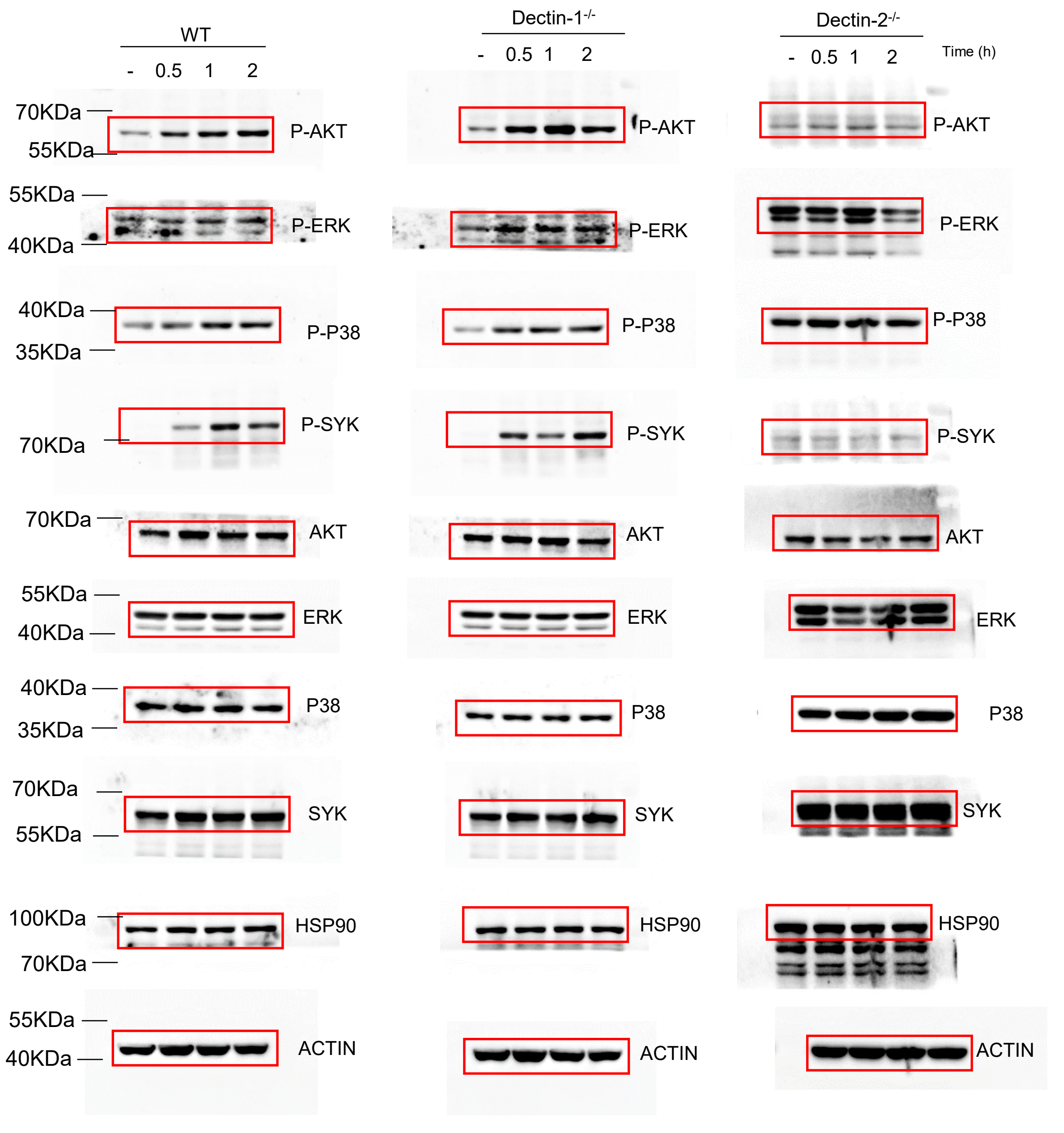
WT
Dectin-1-/-
Dectin-2-/-
- 0.5 1 2
Time (h)
70KDa
55KDa
40KDa
35KDa
100KDa
P-AKT
P-ERK
P-P38
P-SYK
AKT
ERK
P38
SYK
HSP90
ACTIN

Fig. 3e

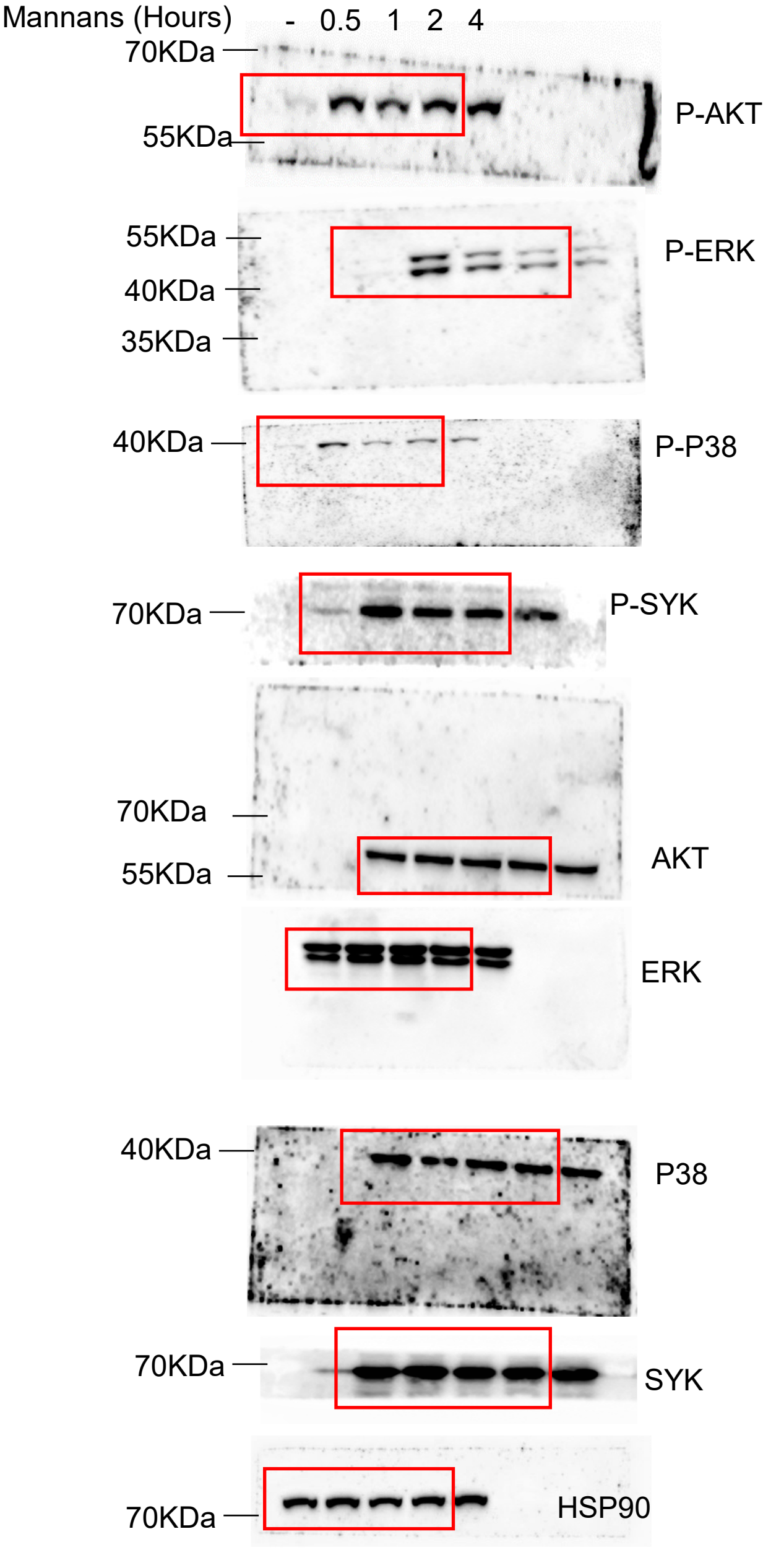
Mannans (Hours)
- 0.5 1 2 4
70KDa
55KDa
P-AKT
55KDa
40KDa
35KDa
P-ERK
40KDa
P-P38
70KDa
P-SYK
70KDa
55KDa
AKT
ERK
40KDa
P38
70KDa
SYK
70KDa
HSP90

Extended Data Fig. 5c

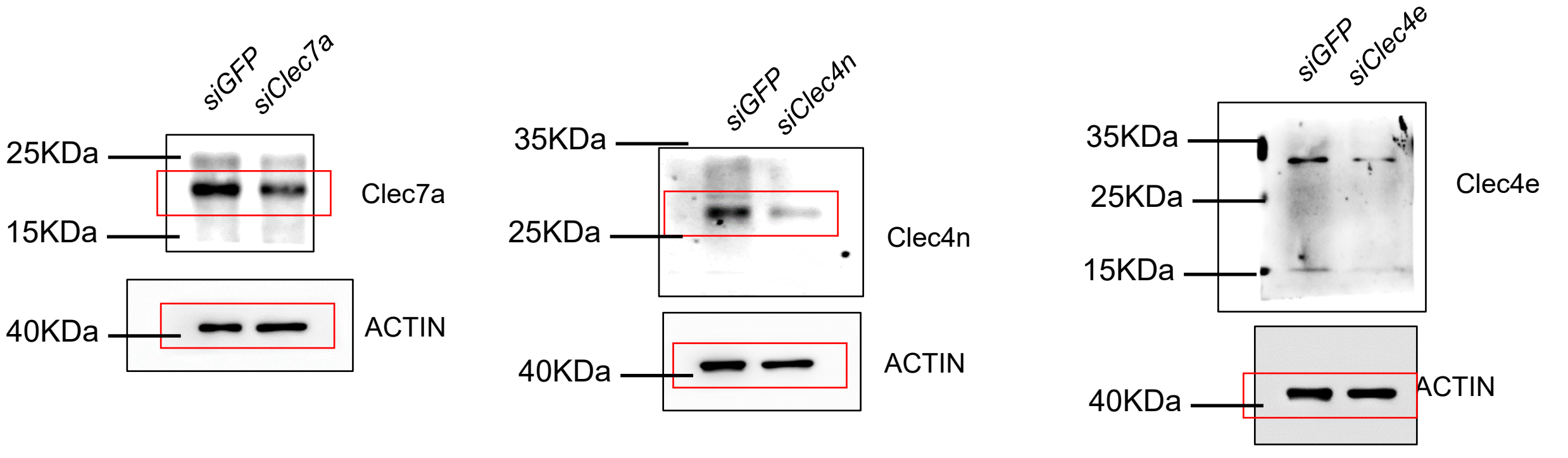

Extended Data Fig. 5d

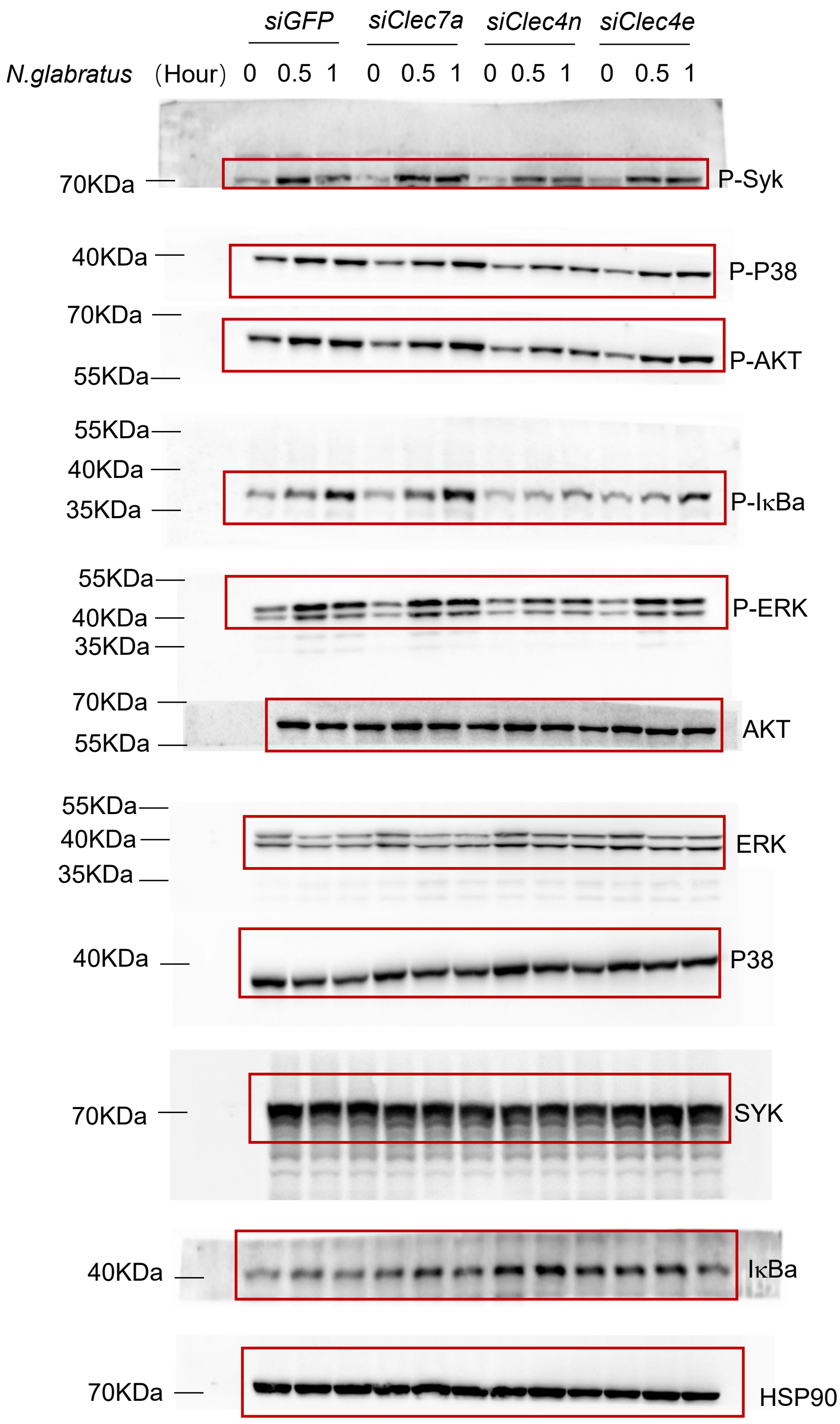

Extended Data Fig. 7g

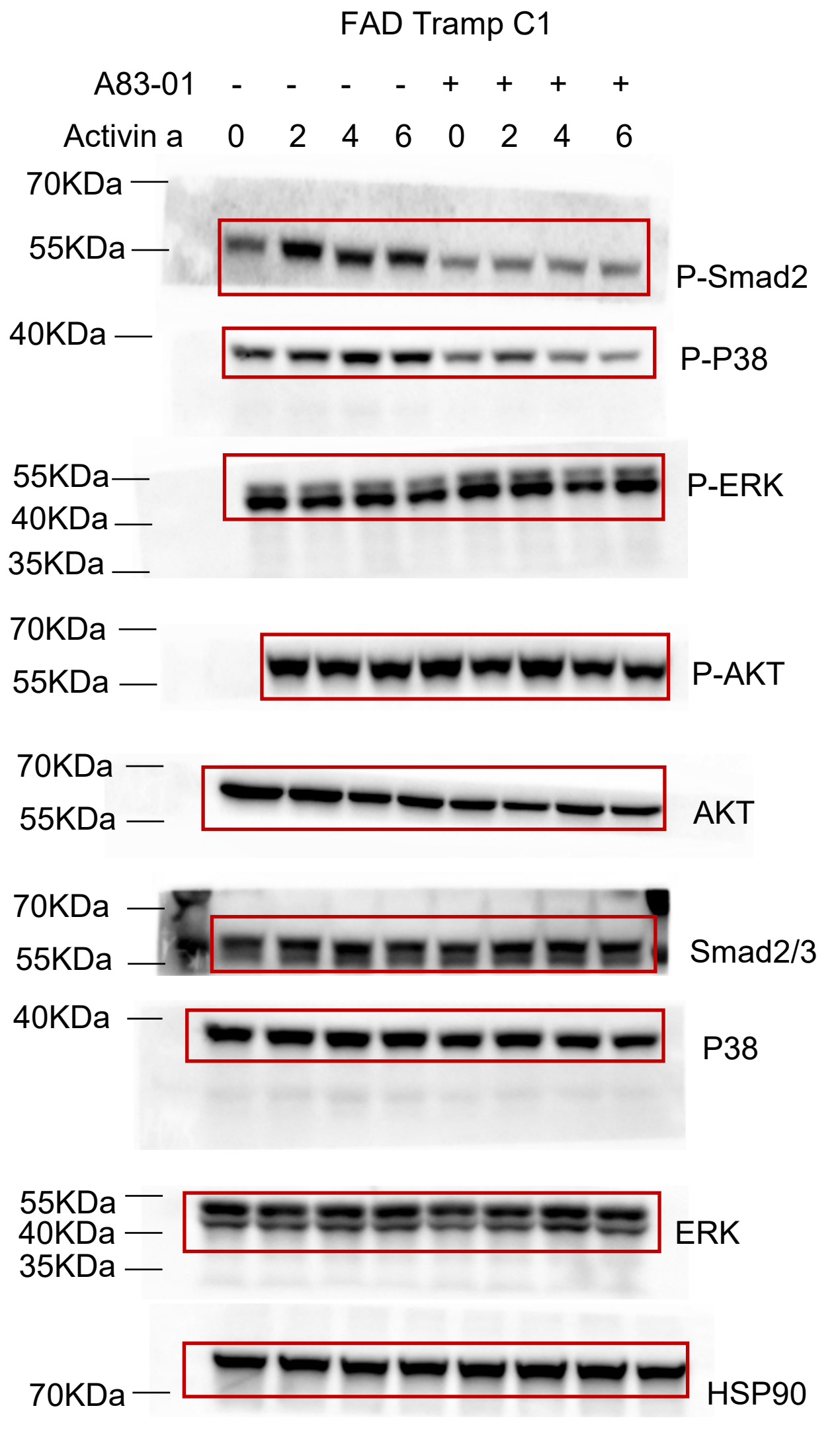

Extended Data Fig. 9a

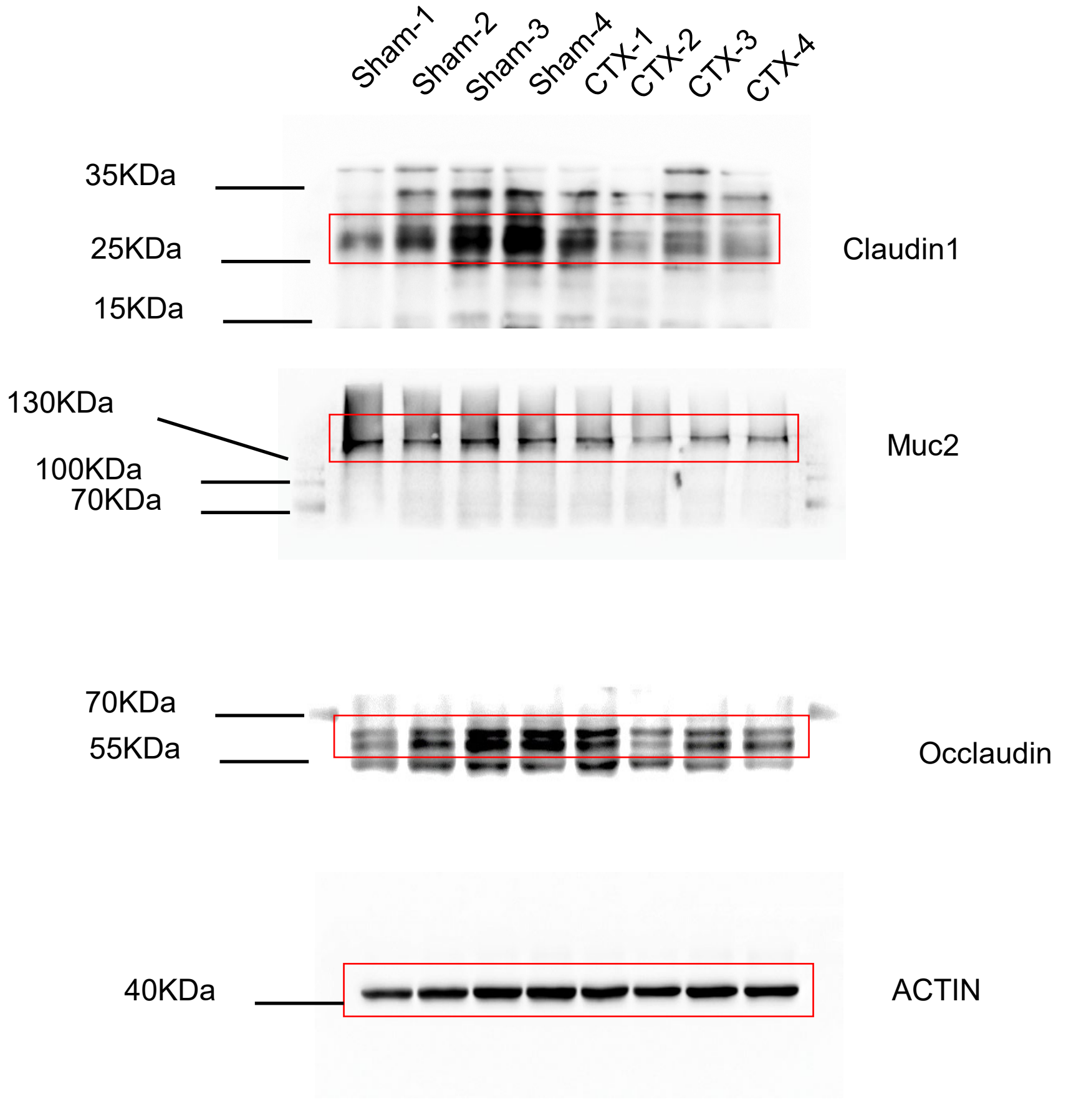

Extended Data Fig. 2e up panel

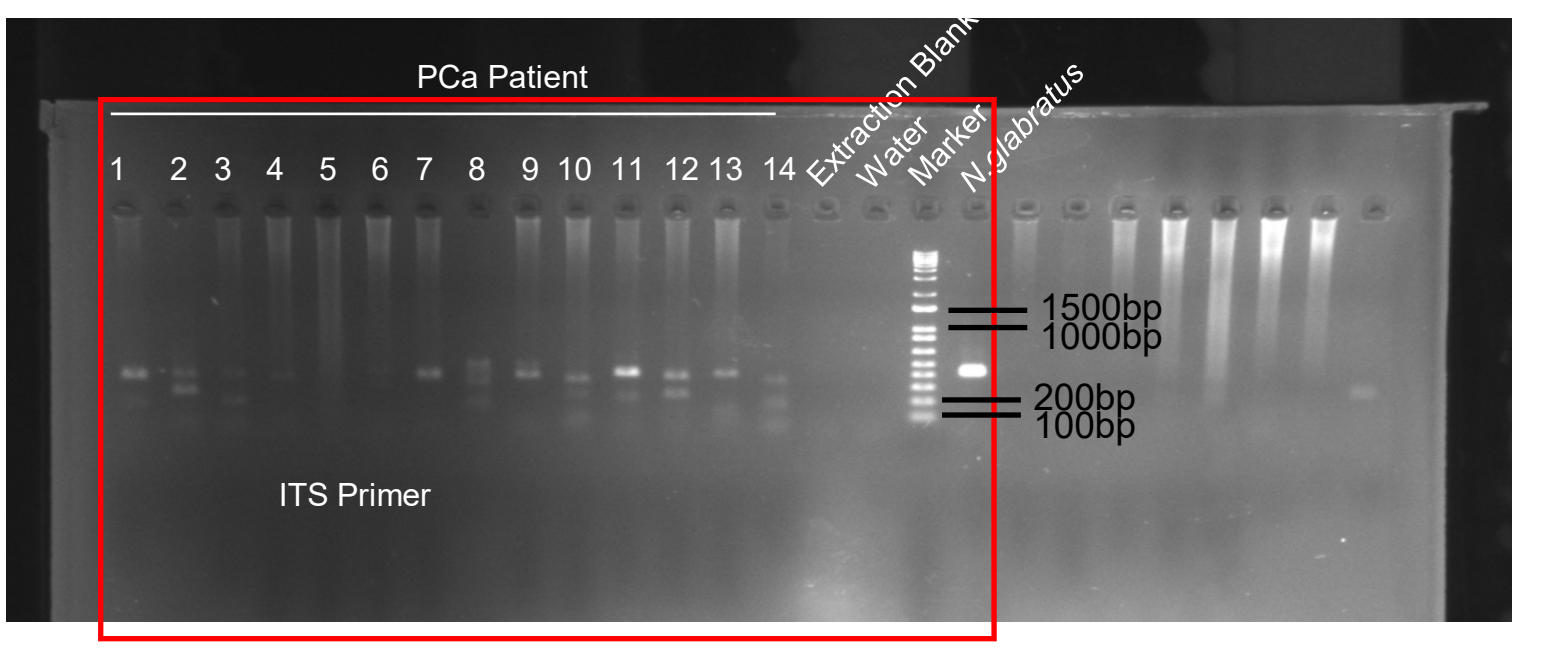

Extended Data Fig. 2e Down panel

Figure 1e

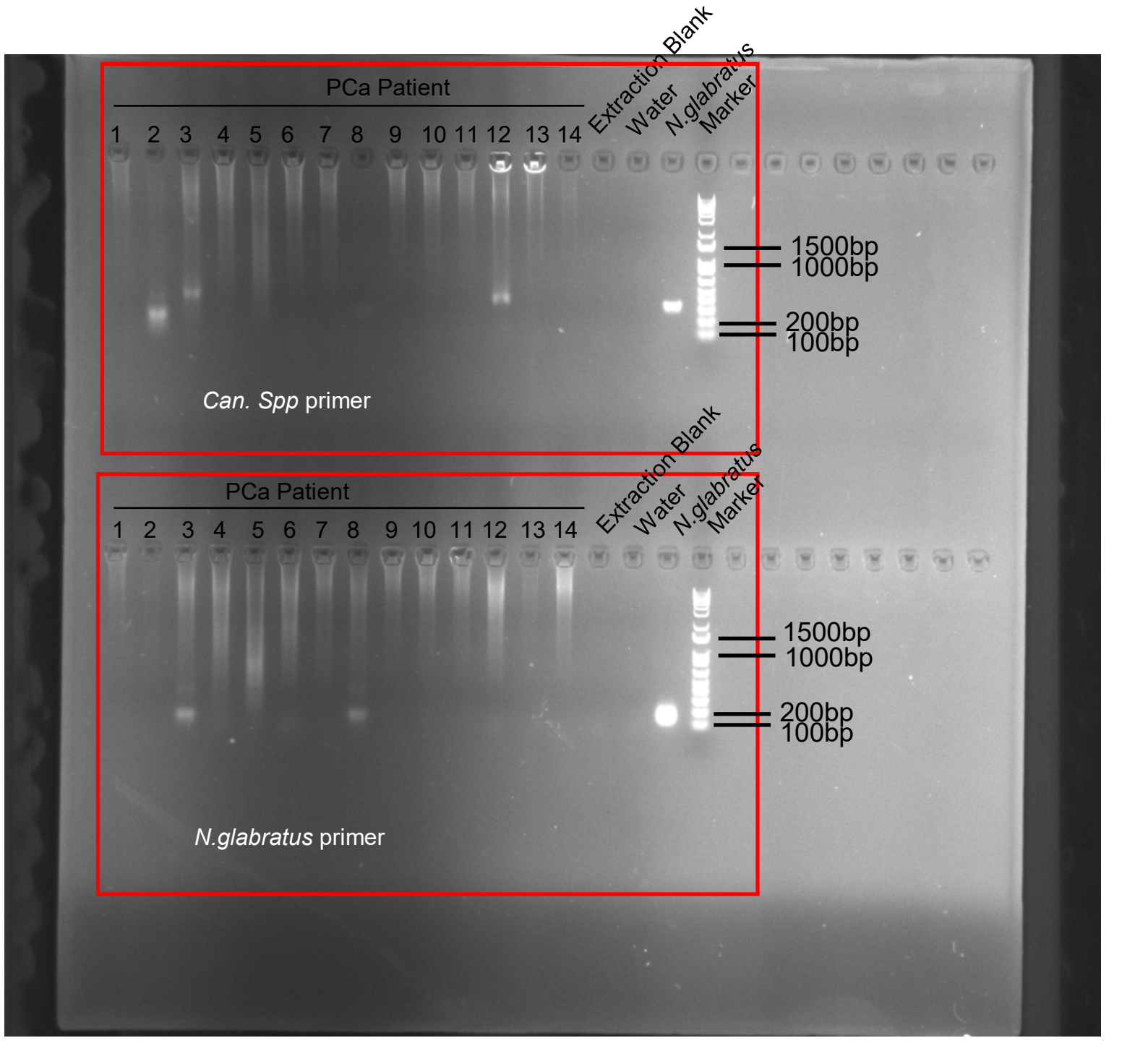

Supplement: Supplementary file 1 [file 43018_2026_1229_MOESM18_ESM.pdf]
