# Supplementary material for: Commensal Nakaseomyces glabratus migrates into prostate tumors to accelerate cancer progression
Source: Nat Cancer. 2026 Sep 9;7(10):1592–609. doi: 10.1038/s43018-026-01229-9 (PMC13694241; doi:10.1038/s43018-026-01229-9)
Supplement: Supplementary file 2 [file 43018_2026_1229_MOESM1_ESM.pdf]

nature cancer

Supplementary information https://doi.org/10.1038/s43018-026-01229-9

Commensal *Nakaseomyces glabratus* migrates into prostate tumors to accelerate cancer progression

In the format provided by the authors and unedited

Supplementary Figure 1

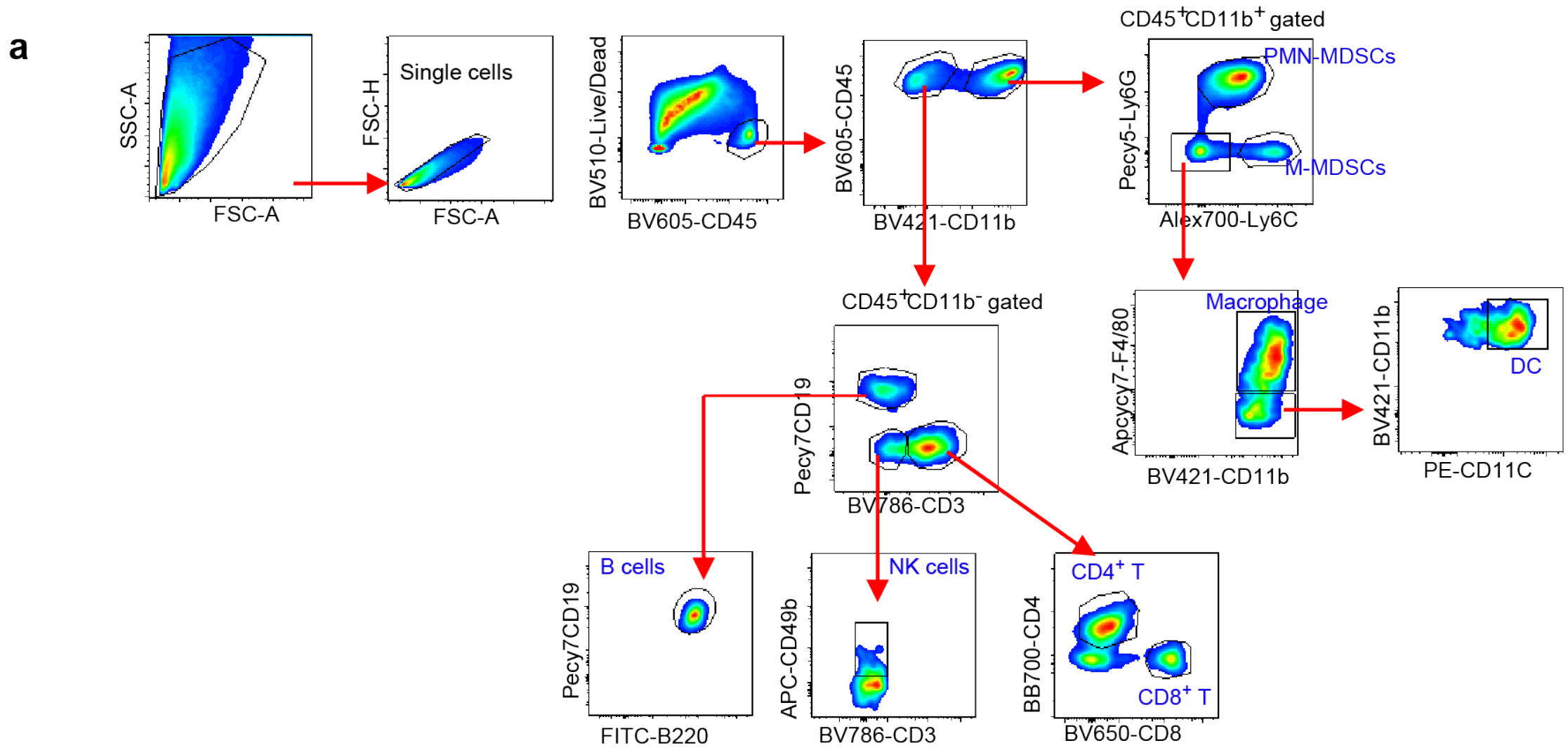

a. Gating strategy of immune cell populations in the tumor microenvironment. The gating strategy is applied in all the other figures unless otherwise specified.
