# Supplementary material for: Commensal Nakaseomyces glabratus migrates into prostate tumors to accelerate cancer progression
Source: Nat Cancer. 2026 Sep 9;7(10):1592–609. doi: 10.1038/s43018-026-01229-9 (PMC13694241; doi:10.1038/s43018-026-01229-9)
Supplement: Supplementary file 3 [file 43018_2026_1229_MOESM2_ESM.pdf]

nature portfolio

Corresponding author(s): Andrea Alimonti
Last updated by author(s): Aug 13, 2026

Reporting Summary

Nature Portfolio wishes to improve the reproducibility of the work that we publish. This form provides structure for consistency and transparency in reporting. For further information on Nature Portfolio policies, see our Editorial Policies and the Editorial Policy Checklist.

Statistics

For all statistical analyses, confirm that the following items are present in the figure legend, table legend, main text, or Methods section.

n/a	Confirmed	
☐	☒	The exact sample size (*n*) for each experimental group/condition, given as a discrete number and unit of measurement
☐	☒	A statement on whether measurements were taken from distinct samples or whether the same sample was measured repeatedly
☐	☒	The statistical test(s) used AND whether they are one- or two-sided<br>*Only common tests should be described solely by name; describe more complex techniques in the Methods section.*
☐	☒	A description of all covariates tested
☐	☒	A description of any assumptions or corrections, such as tests of normality and adjustment for multiple comparisons
☐	☒	A full description of the statistical parameters including central tendency (e.g. means) or other basic estimates (e.g. regression coefficient) AND variation (e.g. standard deviation) or associated estimates of uncertainty (e.g. confidence intervals)
☐	☒	For null hypothesis testing, the test statistic (e.g. *F*, *t*, *r*) with confidence intervals, effect sizes, degrees of freedom and *P* value noted<br>*Give P values as exact values whenever suitable.*
☒	☐	For Bayesian analysis, information on the choice of priors and Markov chain Monte Carlo settings
☒	☐	For hierarchical and complex designs, identification of the appropriate level for tests and full reporting of outcomes
☒	☐	Estimates of effect sizes (e.g. Cohen's *d*, Pearson's *r*), indicating how they were calculated

Our web collection on statistics for biologists contains articles on many of the points above.

Software and code

Policy information about availability of computer code

Data collection	RNA Sequencing: Illumina NextSeq 500/550 (Illumina).<br>Whole-genome metagenome sequencing: Illumina NovaSeq 6000 platform<br>16S rDNA sequencing: Illumina MiSeq<br>ITS sequencign: Illumina NovaSeq 6000 platform<br>Flow cytometry: BD Symphony A5 flow cytometer (BD Biosciences). FACS-sorting: BD FACSAria III.<br>IF image acquisition: Leica TCS SP5.<br>BCA protein quantification: Epoch microplate spectrophotometer.<br>Western blot acquisition: Fusion Solo S.<br>Real-Time PCR: StepOne plus Real-Time PCR system (Applied Biosystem).

Data analysis	Gene expression analysis: STAR (v2.7.10a), DESeq2, ggplot2 v3.3.5., and Pheatmap v1.0.12.<br>Kaplan-Meier curve for disease free survival or overall survival: Tophat2 (v2.0.7), Cufflinks.<br>scRNA seq analysis: CellRanger (v3.1.0), STAR (v2.7.10a). RMagic package, getBM (biomaRt package), Seurat's FindIntegrationAnchors and IntegrateData functions, Seurat's RunTSNE and RunUMAP functions.<br>Whole-genome metagenome analysis: bcl2fastq software (v2.20), FastQC (v0.11.8), DIAMOND (v2.0.8), Kraken2, R package Pavian (v0.8.4).<br>16S rDNA Sequences analysis: DADA2, SILVA reference dataset (v. 138.1), R phyloseq package,<br>ITS sequencing analysis: Usearch (Edgar, 2010), RDP database, SINTAX algorithm.<br>Immunofluorescence: ImageJ, 1.52p, Leica Biosystem.<br>Western blot analysis: ImageJ 1.52p.<br>Flow cytometry analysis: BD FACSDiva software 6.0; FlowJo software 10.1.<br>Data and statistical analysis: GraphPad Prism v10.6.1.

For manuscripts utilizing custom algorithms or software that are central to the research but not yet described in published literature, software must be made available to editors and reviewers. We strongly encourage code deposition in a community repository (e.g. GitHub). See the Nature Portfolio guidelines for submitting code & software for further information.

Data

Policy information about availability of data

All manuscripts must include a data availability statement. This statement should provide the following information, where applicable:

- Accession codes, unique identifiers, or web links for publicly available datasets
- A description of any restrictions on data availability
- For clinical datasets or third party data, please ensure that the statement adheres to our policy

The RNA-seq data of BM-MDSCs was deposited in the Gene Expression Omnibus (GEO) database under accession code GSE311900 (https://www.ncbi.nlm.nih.gov/geo/query/acc.cgi?acc=GSE311900). The 16S sequencing data from mouse fecal samples was deposited in NCBI Sequence Read Archive (SRA) under bioproject ID PRJNA1490896 (https://www.ncbi.nlm.nih.gov/bioproject/1490896). Whole-genome metagenomics data and ITS sequencing data from human fecal swabs generated in this study were deposited in the NCBI SRA database under bioproject ID PRJNA1371343 (https://www.ncbi.nlm.nih.gov/bioproject/?term=PRJNA1371343). The previous publicly available human fecal metagenomics data re-analyzed in this study are available in the NCBI SRA database under bioproject ID PRJNA749645 (https://www.ncbi.nlm.nih.gov/bioproject/?term=PRJNA749645). Previously published scRNA-seq data that were re-analysed here are available under accession code GSE272965 (https://www.ncbi.nlm.nih.gov/geo/query/acc.cgi?acc=GSE272965). Bulk RNA-seq data from the SU2C–PCF cohort were downloaded from https://www.cbioportal.org/study/summary?id=prad_su2c_2019. Bulk RNA-seq data from the Taylor cohort were available in the GEO database under accession code GSE21034 (https://www.ncbi.nlm.nih.gov/geo/query/acc.cgi?acc=GSE21034). The previously published ICR-RMH cohort of the transcriptomic data from fresh mCRPC tissue biopsies is available under restricted access in the European Genome-phenome Archive (EGA) under accession code: EGAS50000001269 (https://ega-archive.org/studies/EGAS50000001269). Access can be requested via EGA. All remaining data supporting the findings of this study are available within the article and its Supplementary Information and/or available upon request from the corresponding author. Source data are provided with this paper.

Research involving human participants, their data, or biological material

Policy information about studies with human participants or human data. See also policy information about sex, gender (identity/presentation), and sexual orientation and race, ethnicity and racism.

Reporting on sex and gender	In this study, all human samples are male.
Reporting on race, ethnicity, or other socially relevant groupings	No special race, ethnicity or other socially relevant groupings.
Population characteristics	The patients cohort (n=31) of Figure 1A for ITS sequencing analysis were from hormone-sensitive PC (n=10) and metastatic CRPC patients (n=21) consented to the analysis of their rectal swabs. Case selection was based on the clinical diagnosis, but independent and blinded to baseline characteristics, clinical outcome and molecular characterization to reduce any potential self-selection bias.<br><br>The patients cohort (n=91) of Figure 1B were metastatic CRPC patients consented to the analysis of their rectal swabs. Case selection was based on the clinical diagnosis, but independent and blinded to baseline characteristics, clinical outcome and molecular characterization to reduce any potential self-selection bias.<br><br>The plasma samples (n= 133) used in Figure 1D were from healthy donors (n=31) and metastatic CRPC patients (n=102).<br><br>The fresh-frozen tumor samples (n= 14) used in Figure 1E were from metastatic CRPC patients .<br><br>The patients data used in Figure 4P, Q, R were the public prostate patients datasets (Taylor, ICR/RMH and SU2C/PCF) which were described in the method section.<br><br>The information of Patient samples used in this study are listed in the Table S1-S4.
Recruitment	Patients were recruited solely on the basis of their survival surgical needs, with no self-selection bias or selection biases introduced in the recruitment process.
Ethics oversight	Blood from healthy donors was obtained from the Swiss Blood Donation Center of Basel and Lugano and used in compliance

Ethics oversight	with the Federal Office of Public Health (authorization no. A000197/2). All prostate cancer patients provided written informed consent and were enrolled in institutional protocols approved by the Royal Marsden Hospital (London, UK) ethics review committee (reference no. 04/Q0801/60) and Ente Ospedaliero Cantonale (EOC) (reference no. CE TI 3269, IOSI-IOR-002; CE TI 3831, IOR-FLO-001), and Padova Province Clinical Experimentation Ethics Committee (reference no. CESC 5170/AO/21).

Note that full information on the approval of the study protocol must also be provided in the manuscript.

Field-specific reporting

Please select the one below that is the best fit for your research. If you are not sure, read the appropriate sections before making your selection.

☒ Life sciences ☐ Behavioural & social sciences ☐ Ecological, evolutionary & environmental sciences

For a reference copy of the document with all sections, see nature.com/documents/nr-reporting-summary-flat.pdf

Life sciences study design

All studies must disclose on these points even when the disclosure is negative.

Sample size	No statistical methods were used to pre-determine sample sizes. Sample sizes were determined based on our previous experience and other similar publication listed in the "Statistic analysis" section.<br>Sample size of human samples was determined by the availability of samples.<br>Sample size of mouse in vivo experiments were estimated based on preliminary experiments and was set to 5-10 mice per group to account for the variability in tumor engraftment. All samples meeting proper experimental conditions were included.<br>Sample size for the in vitro experiments were minimal three biological replicates. The detailed sample sizes are indicated in the figure legends accordingly.
Data exclusions	No data was excluded from the analysis.
Replication	All details were mentioned in the figure legend sections.
Randomization	Animals were randomly assigned to distinct experimental groups and all experimental procedures were implemented in a randomized manner.
Blinding	The investigators were not blinded to group allocation and experimental conditions during experiments and outcome assessment. Data collection and analysis were not performed blind to the conditions of the experiments. Data collection and statistical analysis were conducted objectively without any potential bias.

Reporting for specific materials, systems and methods

We require information from authors about some types of materials, experimental systems and methods used in many studies. Here, indicate whether each material, system or method listed is relevant to your study. If you are not sure if a list item applies to your research, read the appropriate section before selecting a response.

Materials & experimental systems

n/a	Involved in the study	
☐	☒	Antibodies
☐	☒	Eukaryotic cell lines
☒	☐	Palaeontology and archaeology
☐	☒	Animals and other organisms
☒	☐	Clinical data
☒	☐	Dual use research of concern
☒	☐	Plants

Methods

n/a	Involved in the study	
☒	☐	ChIP-seq
☐	☒	Flow cytometry
☒	☐	MRI-based neuroimaging

Antibodies

Antibodies used	Western Blot<br>P-ERK, Cell Signaling Technology, 4370S, 1:1000<br>P-P38, Cell Signaling Technology, 9211S, 1:1000<br>P-AKT, Cell Signaling Technology, 4060S, 1:1000<br>P-IkBa, Cell Signaling Technology 2894S, 1:1000<br>ERK, Cell Signaling Technology, 4695S, 1:1000<br>P38, Cell Signaling Technology, 8690S, 1:1000<br>AKT, Cell Signaling Technology, 9272S, 1:1000<br>IkBa, Cell Signaling Technology, 9242, 1:1000<br>Hsp90, Cell Signaling Technology, 4877S, 1:2000

CLDN1, ThermoFisher, 37-4900, 1:1000
Occludin, ThermoFisher, 40-4700, 1:1000
MUC2, Abcam, ab272692, 1:1000
SYK, Abcam, ab40781, 1:1000
P-SYK, Abcam, ab63515, 1:500
Anti-Rabbit IgG (H+L), HRP Conjugate Promega W4011 1:5000
Anti-MouseIgG(H+L), HRP Conjugate Promega W4021 1:5000
Goat anti-Mouse IgG (H+L), Alexa Fluor Plus 647 ThermoFisher A32728 1:5000
Donkey anti-Rabbit IgG(H&L),Alexa Fluor 647 ThermoFisher A-31573 1:5000

IF and IHC
Ki-67 (clone SP6), Lab Vision Corporation, RM-9106-R7, 1:50
CLDN1, ThermoFisher, 37-4900, 1:200
Occludin, ThermoFisher, 40-4700, 1:200
MUC2, Abcam, ab272692, 1:100

Flow cytometry
Brilliant Violet 605 anti-CD45 (clone 30-F11), Biolegend, 103140, 1:200
Brilliant Violet 421 anti-CD11b (clone M1/70), Biolegend, 101236, 1:200
PE/Cyanine5 anti-Ly-6G (clone 1A8), Thermo Fisher Scientific, 15-9668-82, 1:200
Alexa Fluor(R) 700 anti-Ly-6C (clone HK1.4), Biolegend, 128024, 1:200
PE/Cyanine7 anti-CD19 (clone 1D3), Biolegend, 115520, 1:200
APC anti-CD49b (pan-NK cells) (clone DX5), Biolegend, 108910, 1:200
FITC anti-CD45R/B220 (clone RA3-6B2), Biolegend, 103206, 1:200
Brilliant Violet 650 anti-CD8a (clone 53-6.7), Biolegend, 100742, 1:200
BB700 Rat anti-Mouse CD4 (Clone RM4.5), BD Biosciences, 566407, 1:200
PerCP/Cyanine5.5 anti-CD4 (clone GK1.5), BioLegend, 100434, 1:200
APC/Cyanine7 anti-mouse F4/80 (clone BM8), BioLegend, 123118, 1:200
PE anti-CD11c (clone HL3), BD Biosciences, 553802, 1:200
aCD16/CD32(clone93), BioLegend, 101301, 1:50

In vivo experiment:
InVivoPlus anti-Ly6G antibody (clone 1A8, Bio X Cell, BP0075-1)
InVivoPlus rat IgG2a isotype control (clone 2A3, Bio X Cell, BP0089)

Validation

All antibodies were validated by manufacturers for the specific assay and species used with the validation reports available on the manufacture's website. All antibodies were titrated to determine the optimal working concentration for the detection. Isotope controls were used to gate on cells staining with the antibody of interest.

Eukaryotic cell lines

Policy information about cell lines and Sex and Gender in Research

Cell line source(s)

TRAMP-C1 (CRL-2730) and LNCaP (CRL-1740) cell lines were purchased from ATCC, and B6 Myc-CaP was a kind gift from Prof. Jean-Philippe Theurillat (Institute of Oncology Research, Switzerland). PBMCs were isolated from buffy coats obtained from male healthy donors through the Swiss Blood Donation Center in Basel and Lugano.

Authentication

Cells were previously authenticated by STR fingerprinting prior to their use in the manuscript.

Mycoplasma contamination

Cells were monthly tested for mycoplasma with MycoAlert Mycoplasma Detection kit (Lonza, Cat. LT07-218).

Commonly misidentified lines
(See ICLAC register)

No commonly misidentified cell lines were used in this study.

Animals and other research organisms

Policy information about studies involving animals; ARRIVE guidelines recommended for reporting animal research, and Sex and Gender in Research

Laboratory animals

The Clec4n-/- (C57BL/6J background) mice were obtained from Prof. Yoichiro Iwakura (University of Tokyo). The Clec7a-/- mice, originally constructed by Prof. Gordon Brown (University of Cape Town), were obtained from Prof. Salomé LeibundGut-Landmann (University of Zurich). Inhbaflox/flox mice were generated by Prof. Martin M. Matzuk (Baylor College of Medicine) and then backcrossed to the C57BL/6N background for 10 generations by Prof. Se-jin Lee from The Jackson Laboratory. Lyz2-Cre mice were purchased from the Jackson Laboratory (Strain #: 004781). All mice were propagated under specific-pathogen-free or BSL2 conditions, maintained at a constant temperature of 22±2°C with 50–60% relative humidity and a 12-h light–dark cycle and provided with ad libitum access to standard laboratory chow and sterile drinking water.

Wild animals

No wild animal was used in this study

Reporting on sex

All mice were male.

Field-collected samples	All mice were maintained under specific pathogen-free or BSL2 conditions. Tumor volume were analyzed, and then sacrificed the mice when the tumor of Control group reached approximately 1500 mm3.
Ethics oversight	Experiments were approved by the local ethical committee (''Dipartimento della Sanita` e Socialita`, Esperimenti su animals,'' Canton Ticino), authorization number 34303 and 37779.

Note that full information on the approval of the study protocol must also be provided in the manuscript.

Plants

Seed stocks	*Report on the source of all seed stocks or other plant material used. If applicable, state the seed stock centre and catalogue number. If plant specimens were collected from the field, describe the collection location, date and sampling procedures.*
Novel plant genotypes	*Describe the methods by which all novel plant genotypes were produced. This includes those generated by transgenic approaches, gene editing, chemical/radiation-based mutagenesis and hybridization. For transgenic lines, describe the transformation method, the number of independent lines analyzed and the generation upon which experiments were performed. For gene-edited lines, describe the editor used, the endogenous sequence targeted for editing, the targeting guide RNA sequence (if applicable) and how the editor was applied.*
Authentication	*Describe any authentication procedures for each seed stock used or novel genotype generated. Describe any experiments used to assess the effect of a mutation and, where applicable, how potential secondary effects (e.g. second site T-DNA insertions, mosiacism, off-target gene editing) were examined.*

Flow Cytometry

Plots

Confirm that:

☒ The axis labels state the marker and fluorochrome used (e.g. CD4-FITC).

☒ The axis scales are clearly visible. Include numbers along axes only for bottom left plot of group (a 'group' is an analysis of identical markers).

☒ All plots are contour plots with outliers or pseudocolor plots.

☒ A numerical value for number of cells or percentage (with statistics) is provided.

Methodology

Sample preparation	All details were described in methods and materials.
Instrument	BD FACSymphony A5 and BD FACSAria III
Software	BD FACSDiva software was used for data collection and cell sorting. Data were analyzed using FlowJo software (TreeStar)
Cell population abundance	The purity of post-sorting cells were > 95%
Gating strategy	The gating strategies were presented in Extended data Fig. 3a.

☒ Tick this box to confirm that a figure exemplifying the gating strategy is provided in the Supplementary Information.
